# Socioeconomic diversity of doctors in the United Kingdom: a cross-sectional study of 10 years of Labour Force Survey social mobility data

**DOI:** 10.1136/bmjopen-2024-097178

**Published:** 2025-09-09

**Authors:** Nathan J Cheetham, Fleur Cantle, Andy Guise, Claire J Steves

**Affiliations:** 1 Department of Twin Research and Genetic Epidemiology, King’s College London, London, UK; 2 Department of Emergency Medicine, King’s College Hospital, London, UK; 3 Department of Population Health Sciences, King’s College London, London, UK; 4 Guy’s & St Thomas’s NHS Foundation Trust, London, UK

**Keywords:** Health Workforce, Health Services, MEDICAL EDUCATION & TRAINING

## Abstract

**Abstract:**

**Objectives:**

To estimate the association between socioeconomic background (derived from household main earner occupation when the survey respondent was aged 14 years old) and likelihood of working as a doctor in adulthood in the UK, and estimate how associations varied over time for respondents who turned 18 years old in different decades.

**Design:**

Observational study of 10 years of pooled data from a nationally representative government survey.

**Setting:**

The United Kingdom (UK).

**Participants:**

358 934 respondents to the UK Office for National Statistics Labour Force Survey between July 2014 and September 2023. Respondents aged 22 years old or below or retired respondents aged 65 years old and above were excluded.

**Main outcome measures:**

Whether the respondent was currently working as a medical practitioner (doctor).

**Results:**

2772 respondents were currently working as doctors (0.8% of respondents). 13% of doctors were from working-class backgrounds (National Statistics Socio-economic Classification 5–8), compared with 43% of non-doctor respondents, while 69% of doctors came from professional backgrounds (vs 32% of non-doctors) (unadjusted proportions). From multivariable Poisson regression models adjusting for year of survey, the year the respondent turned 18, sex, country of birth and ethnic group, the likelihood of being a doctor varied largely according to socioeconomic background, with those from professional backgrounds 3 times and 6 times more likely to become doctors than those from intermediate backgrounds and working class backgrounds, respectively (average predicted probability: 1.6% vs 0.5% vs 0.3%). Respondents growing up in households where the main earner was a doctor were by far the most likely to themselves report working as a doctor (average predicted probability: 10.1%), 15-fold more likely than all respondents with non-doctor backgrounds (risk ratio=15.0, 95% CIs 13.4 to 16.7), and between 3 times and 100 times more likely when compared with other specific occupation groups. Stratified analyses suggested socioeconomic inequalities were highly stable over time among respondents who turned 18 between the 1960s and the 2000s, and then weak evidence of decreasing diversity from 2010 to 2018.

**Conclusions:**

There are large, persistent and potentially widening inequalities in the socioeconomic background of doctors working in the UK between 2014 and 2023, leading to doctors being highly socioeconomically unrepresentative of the general UK population. New data collections on the socioeconomic background of working doctors are needed to monitor this inequality and understand its effects on patient care. Increased and/or alternative efforts may be needed to address this entrenched inequality and improve social mobility into medicine.

**STRENGTHS AND LIMITATIONS OF THIS STUDY**

- We pooled 10 years of data from a nationally representative survey to study the socioeconomic background of doctors actively working in the UK.
- We examined how the specific occupation group of the main earner during adolescence is associated with working as a doctor later.
- Stratified analysis by the year the participant turned 18 years old allowed trends in socioeconomic inequality to be estimated across different age cohorts over a 50-year time span.
- Although designed to be nationally representative, the survey data set used here captures only a small fraction (~1%–2%) of the total doctors active between 2014 and 2023.

## Introduction

 Research suggests that doctors are the single most socioeconomically exclusive profession in the UK, with data from 2014 showing that only 4% of practising doctors came from lower-income working-class backgrounds.^1^ Reports highlighting inequalities in the socioeconomic background of medical school students in the UK date back at least to the 1970s and have been a topic of continued focus up to the present day.^2^,^9^ Similar patterns of over-representation of individuals from high-income backgrounds and medical families within medical student populations appear ubiquitous, having been observed internationally in countries across all continents—Europe (the Netherlands,^10 11^ Germany^12^), Asia (Japan^13^), Middle East (Saudi Arabia^14^), North America (Canada,^15^ USA^16^,^18^), South America (Brazil^19^), Africa (Sudan^20^) and Oceania (Australia^21^). Historically, the UK medical profession has exclusive roots dating back hundreds of years, with social hierarchies within medical practitioners and the exclusion of women until the late nineteenth century.^22 23^ Nevertheless, a long-standing belief of the British Medical Association is that ‘doctors should be as representative as possible of the society they serve in order to provide the best possible care to the UK population’.^5 6^ This remains an ongoing explicit aim in the latest workforce plans from the National Health Service (NHS) in England.^24^

Despite these aims, renewed efforts and initiatives in the UK to increase the socioeconomic diversity of medical students over the past 20 years appear to have had little to no effect on admissions, with less privileged students consistently less likely to apply in the first instance and then to be accepted to medical school, based on data from 2007 to 2016.^725^,^27^ When students from under-represented groups do reach medical school, they are more likely to be at the top of their class,^28^ to train to become general practitioners (GPs)^29^,^31^ and to practise in underserved areas that need doctors the most.^32 33^ However, they are also subject to a ‘class pay gap’, receiving lower pay than their counterparts from more privileged backgrounds.^1 34^

Differences in socioeconomic status between doctors and patients potentially impact on the quality of care. Several previous studies in the UK and other countries have found that the patient-doctor relationship is poorer for patients with a lower socioeconomic status, including for studies based on doctors’ own reflections.^35^,^42^ While higher socioeconomic status patients received more information, collaboration, explanation, engagement and humour from doctors, and more control over treatment decisions than their less educated and less affluent counterparts, patients with a lower socioeconomic status were less likely to report receiving enough information to make decisions, and more likely to feel that doctors didn’t give them understandable explanations. Whether the socioeconomic background of doctors itself contributes to these observed inequities in care experiences, either through differences in individual patient-doctor interactions or wider structural health system factors, remains understudied. While a unique environment, a recent study of over a million patient-doctor interactions within the US military explicitly examined the role of the relative status of both patient and doctor, finding that doctors gave more effort and resources to patients ranked above them, and less to those ranked below.^43^

Previous work has examined inequalities in access to undergraduate and postgraduate medical training in the UK for prospective students in the decade from 2007,^7 25^ and snapshots of the socioeconomic backgrounds of medical students in 1966,^3^ 1996–1997,^4^ and doctors working in 2014.^1^ However, none to date have examined how the relationship between socioeconomic background and becoming a doctor has evolved over the past several decades in a single study. Understanding this relationship is essential to understanding progress towards the aim of the medical profession being representative of the society it serves.

In this report, we pooled 10 years of social mobility data collected from 2014 to 2023 as part of the UK Office for National Statistics (ONS) Labour Force Survey (LFS) to examine how parental socioeconomic status and occupation during adolescence affected the likelihood of becoming a doctor. We then probed how associations have varied over time, for those entering medical training from the 1960s to the 2010s.

The research questions of this study were:

1. How does socioeconomic background—from the socioeconomic status of the household main earner during adolescence—as well as the specific main earner occupation, affect the likelihood of working as a doctor as an adult in the UK?
2. How have associations varied over time, based on approximate year of entering medical school (aged 18 years old)?

## Methods

### Data source

Data were drawn from respondents of the UK ONS LFS, a household study of employment circumstances of the UK population.^44^ The LFS is the largest survey of employment in the UK, with around 70 000–90 000 respondents each quarter, and a rolling panel design over five waves, with a fifth entering and exiting at each wave. From 2014, each July–September survey wave has included questions on socioeconomic background. Questions ask about the household composition, main wage earner and occupation of the main wage earner when the respondent was 14 years old.

### Sample selection and outcome variables

Analysis was performed on a pooled data set (n=811 626) comprising annual July–September waves from 2014 to 2023, obtained from the UK Data Service.^45^

To analyse respondents in the working age range of doctors, respondents aged 22 years old or below at the time of survey (n=216 581), or aged 65 years old or above and were retired (n=123 781), were excluded from the pooled data set. Finally, those without socioeconomic background data available were excluded (n=112 330), giving an analysis sample of n=358 934. Age 23 years corresponds to the most common minimum age of a junior doctor following a 5-year medicine undergraduate degree, while age 65 years is the most common state retirement age in the UK. Information on latest occupation prior to retirement was not available in enough detail to identify those who previously worked as doctors.

The outcome variable of interest was whether a respondent was currently employed as a doctor. Current occupation was coded using the Standard Occupational Classification (SOC) (2010 version for 2014–2020 LFS data, 2020 version for 2021–2023 data).^46^ Doctors were identified where SOC2010 unit level code=2211 ‘medical practitioners’ in 2014–2020 data, and where SOC2020 unit level code=2211 ‘generalist medical practitioners’ or 2212 ‘specialist medical practitioners’, in 2021–2023 data.

### Exposure and control variables

The exposures of interest were the occupation and socioeconomic status (derived from occupation) of the main earner of the household when the respondent was aged 14 years old (referred to as the respondent’s socioeconomic background). Main earner occupation was determined using the SOC, with data provided at the minor group level (less detailed than the unit code level available for current occupation). Respondents whose main earners were doctors during adolescence were identified from SOC minor group for 2021–2023 data (SOC2020 minor group code=221, ‘medical practitioners’) and a combination of SOC minor group and the International Standard Classification of Occupations, ISCO-08, minor group for 2014–2020 data (SOC2010 minor group code=221, ‘health professionals’, AND ISCO-08 minor group code=221, ‘medical doctors’). Remaining SOC2010 minor occupation groups for 2014–2020 data were mapped to SOC2020 groups based on ONS mapping files.^47^

Socioeconomic background was determined from the National Statistics Socio-economic Classification (NS-SEC), which itself is derived from the SOC (table 1). Analyses used the 9-class and 3-class versions of the NS-SEC.^48^ We defined a ‘working-class’ background as NS-SEC analytical classes 5–8 as in the 3-class version of the NS-SEC, following UK Government Social Mobility Commission recommendations.^49^ We note this is in contrast to other reports which have defined working class using a narrower definition of NS-SEC 6–8.^1^ Additional variants of NS-SEC were created where doctors were given their own category separate from other NS-SEC groups, to separate the effect of coming from a medical household from other occupations.

**Table 1:** NS-SEC classification of socioeconomic background, derived from occupation of main earner in household when respondents were aged 14 years old, with examples of the two most prevalent occupation groups within each class. Wording for NS-SEC 3-class version is adopted from Social Mobility Commission guidance. The categorisation of doctors within the classification is highlighted in bold.

Socioeconomic background (NS-SEC 3-class)	Socioeconomic background (NS-SEC 9-class)	Example occupation groups (most prevalent SOC2020 minor group)
1–2. Professional background or higher socioeconomic background	1.1. Large employers and higher managerial and administrative occupations	112. Production managers and directors (manufacturing, construction, mining and energy) 113. Functional managers and directors (eg, Sales director, human resources manager)
	1.2. Higher professional occupations	212. Engineering professionals **221. Medical practitioners (doctors)**
	2. Lower managerial, administrative and professional occupations	223. Nursing professionals 231. Teaching professionals
3–4. Intermediate background	3. Intermediate occupations	331. Protective service occupations (eg, officers in police, fire service, prison service) 412. Administrative occupations: finance (eg, payroll manager)
	4. Small employers and own account workers	511. Agricultural and related trades (eg, farmer, fisher, gardener) 531. Construction and building trades
5–8. Working class background or lower socioeconomic background	5. Lower supervisory and technical occupations	522. Metal machining, fitting and instrument making trades (eg, tool maker, air-conditioning installer) 524. Electrical and electronic trades (eg, electrician, telecoms installer)
	6. Semiroutine occupations	711. Sales assistants and retail cashiers 811. Process operatives (eg, food and drink factory machinery operators)
	7. Routine occupations	821. Road transport drivers (eg, bus driver, delivery driver) 913. Elementary process plant occupations (eg, packers, bottlers, cleaners of factory equipment)
	8. Never worked and long-term unemployed	N/A

NS-SEC, National Statistics Socio-economic Classification; SOC2020, Standard Occupational Classification 2020 version.

Control variables were year of survey, year respondent turned 18 years grouped into 5-year bands (derived from year of survey and age in years at time of survey), sex, country of birth, and ethnic group. Age, sex, country of birth and ethnic group were self-reported from closed survey questions. Year respondent turned 18 years was chosen as it represents the age a medicine student most often begins their studies. Response categories for the ethnic group question varied based on the respondent’s nation of residence, and so the least detailed 9-category version common across UK nations was used.

### Weighting

LFS data sets provide individual person weights designed so that the survey can be used to make population estimates that are representative of the age and sex distributions at the regional level.^50^ As individual person weights sum to total UK population estimates, rather than the number of respondents, person weights were scaled linearly downwards to sum to the number of respondents in each year.

To further account for any bias in the missingness of socioeconomic background data (24% missing in the pooled data set), a multivariable logistic regression model was trained based on respondent demographics. A sequential feature selection approach was used to identify the model that maximised predictive power of identifying individuals with missing socioeconomic background data with the fewest variables. The model with the highest area under the receiver operating characteristic curve =0.61 indicating low predictive power, included the following variables: year of survey, year of birth, age at survey, sex, country of birth, ethnic group, UK region of residence, long-term health condition status, highest educational qualification, current economic activity status, and current socioeconomic status (NS-SEC). From this model, weights were derived to represent the inverse probability of having missing socioeconomic background data. Finally, rescaled person weights and newly derived inverse probability weights were multiplied together, producing a weight designed to account for both population representativeness and biases in missing socioeconomic background data.

### Statistical analysis

We used multivariable Poisson regression models with robust errors^51^ to obtain estimates of the total causal effects of the household main earner occupation at age 14 years and socioeconomic background on the outcome of currently being a doctor. To investigate variation in total causal effects estimates over time, further models were run where the analysis sample was stratified based on the year respondents turned 18 years old using a 5-year rolling time window. Secondary analyses estimated associations between the outcome of currently being a doctor and sex, country of birth and ethnic group as individual exposures.

**Figure 1:**
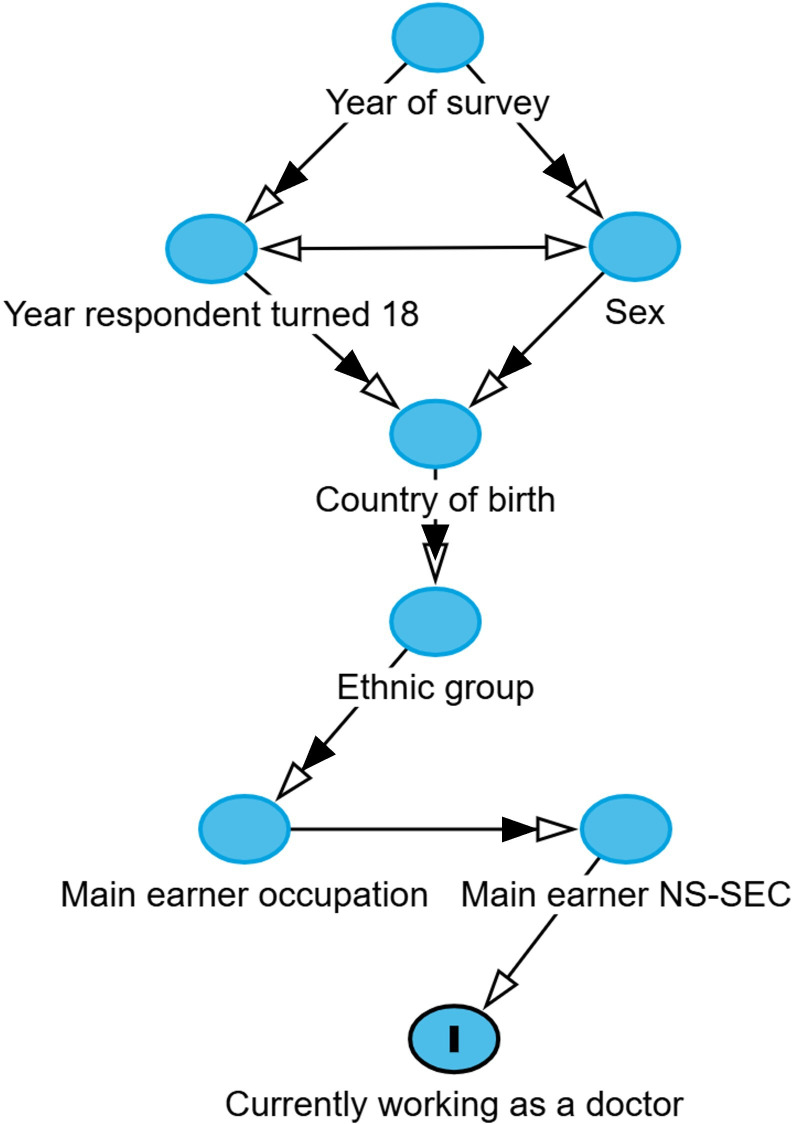
Proposed directed acyclic graph (DAG) used to generate minimal adjustment variable sets for models estimating the total causal effect of exposure variables on the outcome of respondents currently working as a doctor. For clarity, only key ‘nearest neighbour’ arcs are drawn, and double-headed arrows are used to represent where variables are theorised to affect all following variables (ie, arcs present to all following variables). The DAG is structured approximately in order of data generation/crystallisation from top to bottom. NS-SEC, National Statistics Socio-economic Classification.

Separate models were run for each exposure variable. Models included the following potential confounding variables—year the respondent turned 18 years old, sex, country of birth, ethnic group and year of survey—based on the hypothesised directed acyclic graph (DAG) (figure 1). The DAG was developed using DAGitty (http://www.dagitty.net/dags.html),^52^ with full DAGitty code given in the data supplement. Models used the ‘HC3’ estimator of coefficient standard errors to account for heteroskedasticity.^53^

Adjusted mean predicted probabilities of being a doctor were generated from regression model fits following an ‘average marginal effects’ or ‘marginal standardisation’ approach,^54^ using the ‘marginaleffects’ R package.^55^ In this approach, individual-level predicted probabilities were generated after assigning all individuals to a single category of the exposure variable, while keeping all other model variables at their observed values. The mean of all predicted probabilities was then calculated, while accounting for inverse probability weights. This process was repeated while varying the exposure variable value, thereby generating a mean predicted probability for all exposure categories. Again, 95% confidence intervals (CIs) were calculated using the ‘HC3’ estimator.

The Python analysis script used to produce results is provided at https://github.com/nathan-cheetham/LFSDoctorSocioeconomicBackground.

### Software

Poisson regression models were fit and figures were created using Python V.3.8.8 and packages: numpy V.1.20.1, pandas V.1.2.4, statsmodels V.0.12.2, scipy V.1.6.2, scikit-learn V.0.24.1, matplotlib V.3.3.4, seaborn V.0.11.1. Mean predicted probabilities were calculated using R V.4.3.0 and packages: haven V.2.5.4, tidyverse V.2.0.0, ggeffects V.1.5.2, lme4 V.1.1.35.2, merTools V.0.6.2, labelled V.2.13.0, sjPlot V.2.8.16, Metrics V.0.1.4, dplyr V.1.1.4, marginaleffects_0.25.0, sandwich_3.1–1.

### Patient and public involvement

No patients or members of the public were directly involved in this study.

## Results

### Sample characteristics

The analysis sample comprised 358 934 respondents with socioeconomic background data available, aged between 23 years and 64 years old or 65 years old and above and not retired at the time of survey. Of these, 2772 (0.8%) respondents were currently working as medical practitioners (referred to throughout as doctors).

Respondents working as doctors varied in their distributions of age, country of birth, ethnic group and socioeconomic background versus all other respondents (table 2). Socioeconomic background was derived from the occupation of the household main earner when respondents were aged 14 years old, with details of the NS-SEC classification and example occupations given in table 1. Doctors were younger than non-doctor respondents, with larger unadjusted proportions of doctors from non-UK countries of birth compared with non-doctor respondents (37% vs 18%), and racially minoritised ethnic groups (35% vs 13%). A much higher proportion of doctors was from professional backgrounds compared with non-doctors (69% vs 33%), and lower proportions of doctors originated from intermediate backgrounds (18% vs 24%) and working class backgrounds (13% vs 43%). Around 1 in 7, 14%, of respondents who came from a household where the main earner was a doctor were currently working as a doctor, versus 0.8% in the whole sample.

**Table 2:** Sample characteristics

Variable	Current doctors	% doctors (as proportion of all current doctors)	% doctors (as proportion of respondents)	All other respondents	% other respondents (as proportion of all other respondents)	Total	% total (as proportion of total)
Total	**2772**	**100%**	**0.8%**	**356 162**		**358 934**	
Age (median, IQR), years	42 (34–52)			48 (36–58)		48 (36–58)	
Year respondent turned 18 years old							
Pre-1970	61	2.2%	0.5%	12 178	3.4%	12 239	3.4%
1970–1974	85	3.1%	0.4%	23 814	6.7%	23 899	6.7%
1975–1979	168	6.1%	0.4%	38 965	10.9%	39 133	10.9%
1980–1984	273	9.8%	0.6%	44 747	12.6%	45 020	12.5%
1985–1989	287	10.4%	0.6%	44 042	12.4%	44 330	12.4%
1990–1994	361	13.0%	0.9%	39 552	11.1%	39 914	11.1%
1995–1999	404	14.6%	1.0%	39 485	11.1%	39 889	11.1%
2000–2004	470	16.9%	1.1%	40 968	11.5%	41 437	11.5%
2005–2009	431	15.5%	1.0%	40 986	11.5%	41 417	11.5%
Post-2010	233	8.4%	0.7%	31 424	8.8%	31 656	8.8%
Sex							
Female	1392	50.2%	0.8%	179 819	50.5%	181 211	50.5%
Male	1380	49.8%	0.8%	176 343	49.5%	177 723	49.5%
Country of birth							
UK nation	1756	63.3%	0.6%	293 102	82.3%	294 858	82.1%
England	1397	50.4%	0.6%	240 323	67.5%	241 721	67.3%
Northern Ireland	99	3.6%	0.8%	11 605	3.3%	11 704	3.3%
Scotland	177	6.4%	0.7%	26 920	7.6%	27 097	7.5%
Wales	81	2.9%	0.6%	14 010	3.9%	14 091	3.9%
Unspecified UK nation	<5	0.0%	0.5%	244	0.1%	245	0.1%
Non-UK	1016	36.7%	1.6%	63 059	17.7%	64 076	17.9%
India	228	8.2%	4.0%	5467	1.5%	5695	1.6%
Pakistan	75	2.7%	2.2%	3371	0.9%	3446	1.0%
Poland	19	0.7%	0.3%	5430	1.5%	5448	1.5%
Ireland	24	0.9%	1.4%	1669	0.5%	1693	0.5%
Any other country	670	24.2%	1.4%	47 123	13.2%	47 793	13.3%
Ethnic group							
Racially minoritised ethnic groups	979	35.3%	2.1%	45 019	12.6%	45 998	12.8%
Asian/Asian British ethnic groups, Bangladeshi	35	1.3%	1.4%	2434	0.7%	2469	0.7%
Asian/Asian British ethnic groups, Chinese	51	1.8%	2.3%	2186	0.6%	2237	0.6%
Asian/Asian British ethnic groups, Indian	342	12.3%	3.4%	9770	2.7%	10 112	2.8%
Asian/Asian British ethnic groups, Pakistani	132	4.8%	2.3%	5708	1.6%	5840	1.6%
Asian/Asian British ethnic groups, any other	99	3.6%	2.2%	4353	1.2%	4451	1.2%
Black/Black British ethnic groups	110	4.0%	1.0%	10 676	3.0%	10 786	3.0%
Mixed/Multiple ethnic groups	70	2.5%	1.7%	3972	1.1%	4042	1.1%
Any other ethnic groups	140	5.1%	2.3%	5920	1.7%	6060	1.7%
White ethnic groups	1791	64.6%	0.6%	310 916	87.3%	312 707	87.1%
Missing	<5	0.1%	1.2%	227	0.1%	230	0.1%
Socioeconomic background							
NS-SEC 1–2. Professional backgrounds	1905	68.7%	1.6%	115 595	32.5%	117 500	32.7%
1.1. Large employers and higher managerial and administrative occupations	229	8.3%	1.2%	18 397	5.2%	18 626	5.2%
1.2. Higher professional occupations	1024	36.9%	2.7%	36 910	10.4%	37 933	10.6%
1.2. Medical practitioners (doctors)	453	16.3%	13.8%	2839	0.8%	3292	0.9%
1.2. All other higher professional occupations	571	20.6%	1.6%	34 071	9.6%	34 642	9.7%
2. Lower managerial, administrative and professional occupations	652	23.5%	1.1%	60 289	16.9%	60 941	17.0%
NS-SEC 3–4. Intermediate backgrounds	498	17.9%	0.6%	85 835	24.1%	86 333	24.1%
3. Intermediate occupations	286	10.3%	0.8%	36 256	10.2%	36 542	10.2%
4. Small employers and own account workers	212	7.6%	0.4%	49 579	13.9%	49 791	13.9%
NS-SEC 5–8. Working class backgrounds (including not living with family)	370	13.3%	0.2%	154 732	43.4%	155 101	43.2%
5. Lower supervisory and technical occupations	128	4.6%	0.3%	38 019	10.7%	38 147	10.6%
6. Semiroutine occupations	85	3.1%	0.2%	38 442	10.8%	38 527	10.7%
7. Routine occupations	99	3.6%	0.2%	57 850	16.2%	57 949	16.1%
8. Never worked and long-term unemployed	40	1.5%	0.2%	16 657	4.7%	16 698	4.7%
Not living with family	18	0.6%	0.5%	3762	1.1%	3780	1.1%
Survey year							
2014	228	8.2%	0.6%	36 590	10.3%	36 818	10.3%
2015	264	9.5%	0.7%	36 680	10.3%	36 944	10.3%
2016	299	10.8%	0.8%	36 829	10.3%	37 128	10.3%
2017	246	8.9%	0.7%	37 101	10.4%	37 346	10.4%
2018	248	8.9%	0.7%	37 149	10.4%	37 397	10.4%
2019	278	10.0%	0.7%	37 426	10.5%	37 704	10.5%
2020	313	11.3%	0.9%	35 722	10.0%	36 035	10.0%
2021	364	13.1%	1.0%	36 757	10.3%	37 121	10.3%
2022	341	12.3%	1.0%	35 049	9.8%	35 390	9.9%
2023	193	7.0%	0.7%	26 858	7.5%	27 051	7.5%

Counts from sum of individual weights, accounting for population age and sex distribution, and likelihood of socioeconomic background data availability. Weighted counts of less than 5 are masked.

NS-SEC, National Statistics Socio-economic Classification.

### Descriptive trends in socioeconomic background

**Figure 2:**
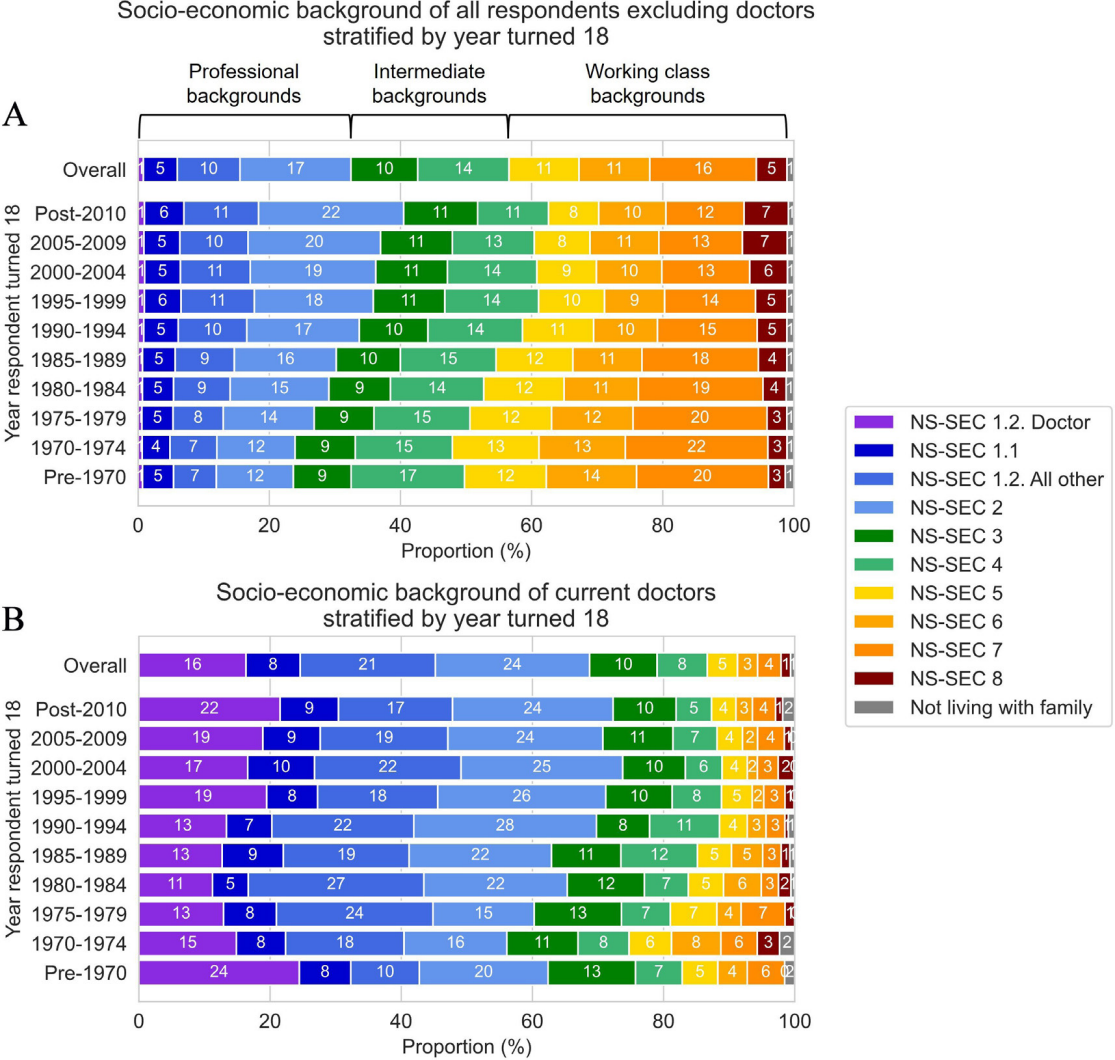
(**A**) Unadjusted socioeconomic background of all survey respondents (age 23–64 years or 65+ years and not retired) excluding current doctors (n=356 162) from household main earner NS-SEC at age 14 years. (**B**) Unadjusted socioeconomic background of current doctors (n=2772). Proportions derived from the sum of individual weights. NS-SEC, National Statistics Socio-economic Classification.

Plotting socioeconomic background (based on household main earner occupation at age 14 years) versus year respondent turned 18 years old shows the reduction of working class occupations and growth of professional roles over time in the UK population, from figures for the 99.2% of respondents who were not currently working as doctors (figure 2A). Working-class backgrounds declined from 51% for those who turned 18 years old in 1970–1974 (born in the early mid-1950s) to 37% for those turning 18 years old in the 2010s (born in the 1990s). In contrast, the prevalence of professional backgrounds increased from 24% for respondents entering adulthood in the early 1970s to 40% by the 2010s.

In contrast to the general population, a much higher proportion of respondents who were working as doctors came from professional backgrounds (figure 2B), with particularly large proportions coming from households where the main earner was a doctor (16%). Doctors also showed smaller trends over time, with the proportion from professional backgrounds increasing from approximately 60% to 70% between the 1970s and 2010s, and the proportion from working class backgrounds decreasing from approximately 20% to 10% between the 1970s and 2010s.

### Associations between working as a doctor and socioeconomic background and occupation from multivariable models

**Figure 3:**
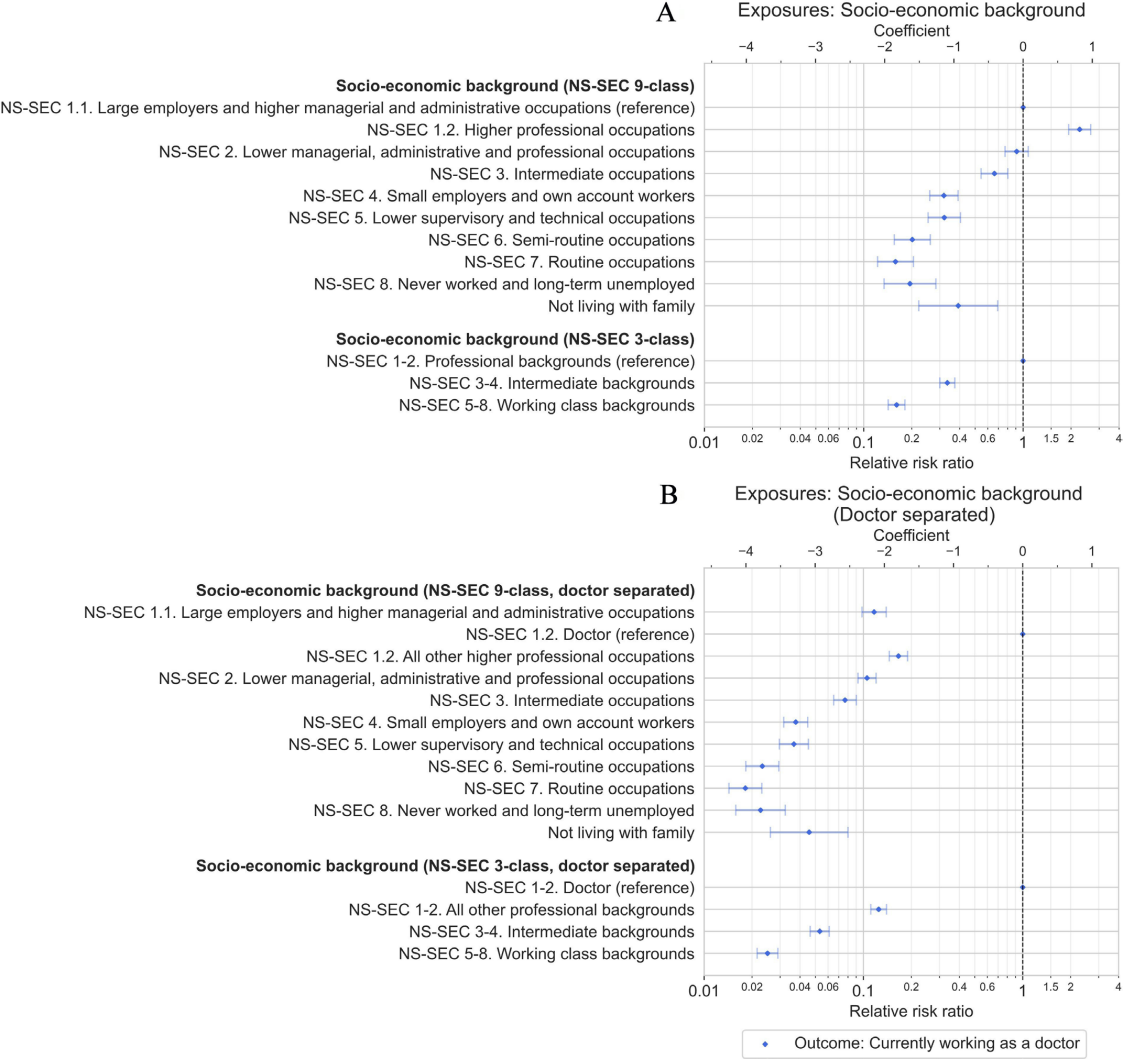
Relative risk ratios with 95% CIs from Poisson regression models estimating the association between socioeconomic background and working as a doctor. Models adjusted for year respondent turned 18 years, sex, country of birth, ethnic group and year of survey. Socioeconomic background is modelled using 9-class and 3-class versions of the NS-SEC derived from household main earner occupation at age 14 years, without (A), and with (B), separate categories to identify where household main earner was a doctor. NS-SEC, National Statistics Socio-economic Classification.

Strong associations between socioeconomic background and currently working as a doctor were observed in multivariable Poisson regression models (figure 3 and table 3). Models controlled for year respondent turned 18 years old to control for trends in the relative shares of different occupations over time, as well as sex, country of birth, ethnic group and year of survey response.

**Table 3:** Summary of unadjusted weighted counts and adjusted results from multivariable Poisson regression models estimating the association between socioeconomic background and working as a doctor

Socioeconomic background	Unadjusted counts (weighted)				Adjusted mean predicted probability	Adjusted risk ratio (95% CI)			
	Respondents	Current doctors	% respondents	% doctors	Probability (95% CI)	Model 1	Model 2	Model 3	Model 4
NS-SEC 1–2. Professional backgrounds (m1)	117 500	1905	1.6%	68.7%	1.6% (1.5% to 1.7%)	1 (ref.)			
1.1. Large employers and higher managerial and administrative occupations	18 626	229	1.2%	8.3%	1.2% (1.0% to 1.3%)		1 (ref.)		0.12 (0.10 to 0.14)
1.2. Higher professional occupations (m2)	37 933	1024	2.7%	36.9%	2.6% (2.5% to 2.8%)		2.26 (1.93 to 2.66)		
1.2. Medical practitioners (doctors)	3292	453	13.8%	16.3%	10.1% (9.1% to 11.1%)			1 (ref.)	1 (ref.)
1.2. All other higher professional occupations	34 642	571	1.6%	20.6%	1.7% (1.5% to 1.8%)				0.17 (0.15 to 0.19)
2. Lower managerial, administrative and professional occupations	60 941	652	1.1%	23.5%	1.1% (1.0% to 1.2%)		0.91 (0.77 to 1.08)		0.11 (0.09 to 0.12)
1–2. Professional backgrounds excluding doctors (m3)	114 209	1452	1.3%	52.4%	1.3% (1.2% to 1.3%)			0.12 (0.11 to 0.14)	
NS-SEC 3–4. Intermediate backgrounds (m1)	86 333	498	0.6%	17.9%	0.5% (0.5% to 0.6%)	0.33 (0.30 to 0.37)		0.05 (0.05 to 0.06)	
3. Intermediate occupations	36 542	286	0.8%	10.3%	0.8% (0.7% to 0.9%)		0.66 (0.54 to 0.8)		0.08 (0.07 to 0.09)
4. Small employers and own account workers	49 791	212	0.4%	7.6%	0.4% (0.3% to 0.4%)		0.32 (0.26 to 0.39)		0.04 (0.03 to 0.04)
NS-SEC 5–8. Working class backgrounds (including not living with family) (m1)	155 101	370	0.2%	13.3%	0.3% (0.2% to 0.3%)	0.16 (0.14 to 0.18)		0.03 (0.02 to 0.03)	
5. Lower supervisory and technical occupations	38 147	128	0.3%	4.6%	0.4% (0.3% to 0.4%)		0.32 (0.25 to 0.40)		0.04 (0.03 to 0.05)
6. Semiroutine occupations	38 527	85	0.2%	3.1%	0.2% (0.2% to 0.3%)		0.20 (0.16 to 0.26)		0.02 (0.02 to 0.03)
7. Routine occupations	57 949	99	0.2%	3.6%	0.2% (0.1% to 0.2%)		0.16 (0.12 to 0.20)		0.02 (0.01 to 0.02)
8. Never worked and long-term unemployed	16 698	40	0.2%	1.5%	0.2% (0.1% to 0.3%)		0.20 (0.13 to 0.28)		0.02 (0.02 to 0.03)
Not living with family	3780	18	0.5%	0.6%	0.5% (0.2% to 0.7%)		0.39 (0.22 to 0.69)		0.05 (0.03 to 0.08)
Total	358 934	2772	0.8%	100%					

Multivariable models adjusted for year respondent turned 18 years old, sex, country of birth, ethnic group and year of survey. Model 1 modelled NS-SEC as 3-class variable, not separating doctor, with reference category: NS-SEC 1–2. Model 2 modelled NS-SEC as 9-class variable, not separating doctor, with reference category: NS-SEC 1.1. Model 3 modelled NS-SEC as 3-class variable, with doctor as a separated category, with reference category: Doctor. Model 4 modelled NS-SEC as 9-class variable, with doctor as a separated category, with reference category: Doctor. Unless otherwise stated in category name, adjusted mean predicted probabilities results are presented from model 4.

NS-SEC, National Statistics Socio-economic Classification.

The effect size closely followed the ordering of the NS-SEC analytical classes (from 1 to 8), which are correlated with the average income of the occupations within,^56^ with likelihood of working as a doctor highest among professional backgrounds (NS-SEC 1–2) and lowest among working class backgrounds (NS-SEC 5–8). Compared with respondents from professional backgrounds (NS-SEC 1–2), those from intermediate backgrounds (NS-SEC 3–4) were threefold less likely to be doctors (risk ratio, RR=0.33, 95% CI 0.30 to 0.37), and those from working class backgrounds (NS-SEC 5–8) were sixfold less likely (RR=0.16, 95% CI 0.14 to 0.18) (figure 3A). The occupational class for which being a doctor was most likely was NS-SEC 1.2 ‘Higher professional occupations’ (mean predicted probability: 2.6%, 95% CI 2.5% to 2.8%), which contains doctors (respondents where the main earner at age 14 years was a doctor made up 9% of NS-SEC 1.2 and 3% of NS-SEC 1–2 in the sample).

Models where doctors were separated from the rest of the occupational class and acted as the reference category found even larger inequalities (figure 3B). While the mean predicted probability of being a current doctor was 10.1% (95% CI 9.1% to 11.1%) for those who came from doctor households, the probability was only 0.3% (95% CI 0.2% to 0.3%) for respondents from working-class households, a 40-fold difference (RR=0.025, 95% CI 0.022 to 0.029). Large inequalities were even found for those with the most privileged non-doctor backgrounds, with respondents coming from households working in other professional occupations still eightfold less likely to be doctors versus those from doctor backgrounds (RR=0.12, 95% CI 0.11 to 0.14).

Inequalities were also observed in models estimating associations with sex, country of birth and ethnic group (online supplemental figure S1), with respondents born in several non-UK countries more likely than those born in England to be doctors, and those of Asian/Asian British, Black/Black British, Mixed/Multiple and any other ethnic groups all more likely to be doctors than those of White ethnic groups.

**Figure 4:**
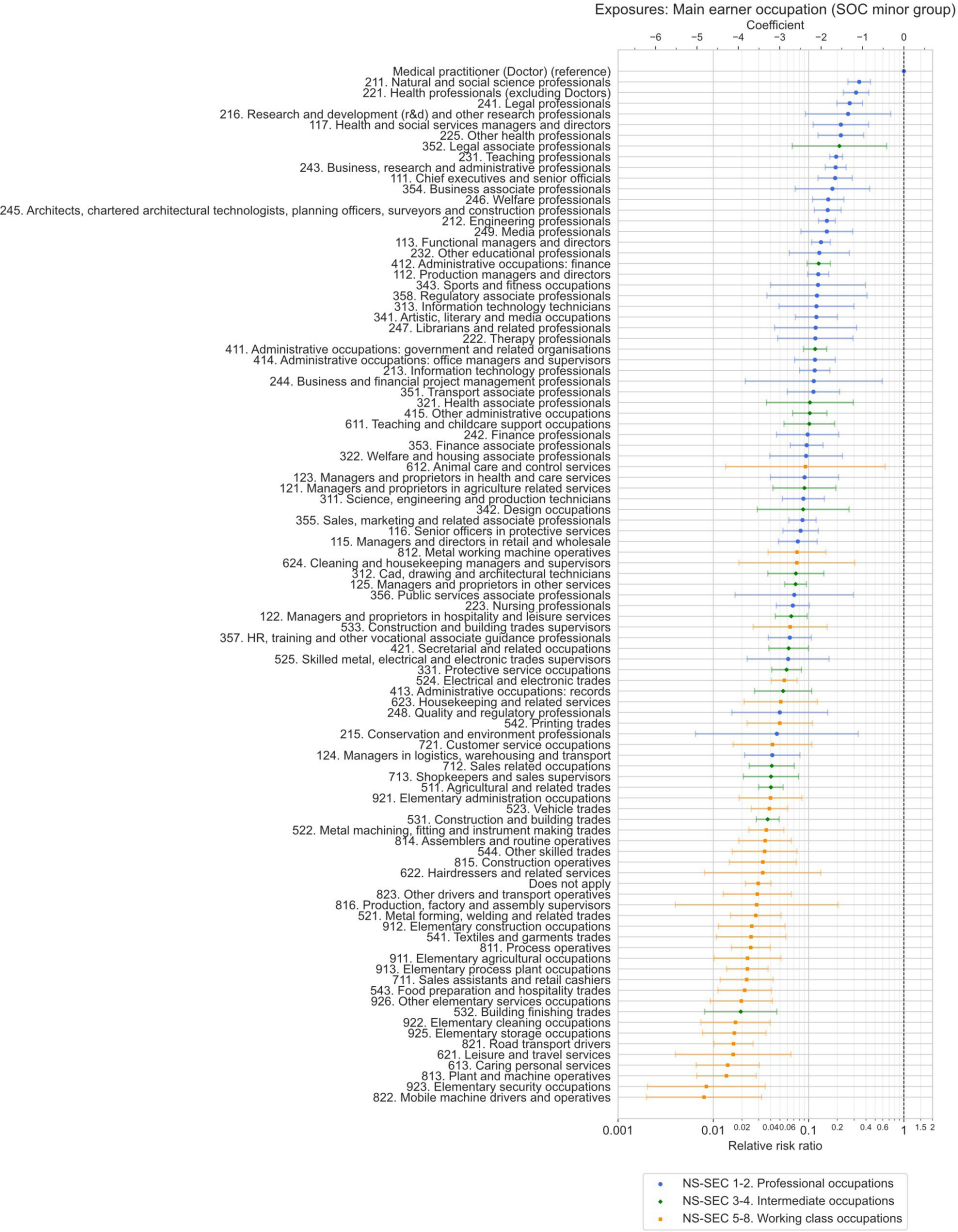
Relative risk ratios with 95% CIs from Poisson regression models estimating the association between main earner occupation group (SOC2020 minor group) at age 14 years and working as a doctor, adjusting for year turned 18 years, sex, country of birth, ethnic group and year of survey. Doctors acted as the reference group. Occupation groups are colour coded according to most common NS-SEC category (3-class version) for the given occupation group. NS-SEC, National Statistics Socio-economic Classification; SOC2020, Standard Occupational Classification 2020 version.

The likelihood of being a doctor based on the household main earner’s specific SOC2020 minor occupation group at age 14 years was also estimated (figure 4, table 4 and online supplemental table S1 for all occupations). Of 106 occupation groups, 27 of the 30 lowest predicted probability groups were working-class occupations, while 28 of the 31 highest probability groups were professional occupations. Just over half of the doctors in the sample came from the 17 main earner occupation groups with highest predicted probabilities—1414 doctors from 58 569 respondents—with the remaining 1358 doctors coming from 300 365 respondents in the 89 occupation groups with the lowest predicted probabilities.

**Table 4:** Main earner occupations (SOC2020 minor groups) of respondents with highest and lowest mean predicted probabilities of being a doctor

Main earner occupation (SOC2020 minor group)	NS-SEC (9-class)	Unadjusted counts (weighted)				Adjusted mean predicted probability (95% CI)	Adjusted risk ratio (95% CI)
		Current doctors	Respondents	% respondents	% doctors		
**Ten least likely occupations**							
822. Mobile machine drivers and operatives	6	<5	2275	0.06%	0.1%	0.07% (0% to 0.17%)	0.008 (0.002 to 0.032)
923. Elementary security occupations	6	<5	2374	0.07%	0.1%	0.08% (0% to 0.19%)	0.008 (0.002 to 0.035)
813. Plant and machine operatives	7	8	7757	0.10%	0.3%	0.12% (0.04% to 0.21%)	0.014 (0.007 to 0.028)
613. Caring personal services	6	7	5381	0.12%	0.2%	0.13% (0.03% to 0.23%)	0.014 (0.007 to 0.030)
621. Leisure and travel services	6	<5	1253	0.16%	0.1%	0.15% (0% to 0.35%)	0.016 (0.004 to 0.065)
821. Road transport drivers	7	22	15 502	0.14%	0.8%	0.15% (0.08% to 0.22%)	0.016 (0.010 to 0.026)
925. Elementary storage occupations	7	6	4289	0.13%	0.2%	0.15% (0.04% to 0.27%)	0.017 (0.008 to 0.036)
922. Elementary cleaning occupations	7	8	5878	0.14%	0.3%	0.16% (0.03% to 0.28%)	0.017 (0.007 to 0.039)
532. Building finishing trades	4	7	4595	0.14%	0.2%	0.18% (0.02% to 0.33%)	0.019 (0.008 to 0.046)
926. Other elementary services occupations	7	7	3771	0.18%	0.2%	0.18% (0.05% to 0.31%)	0.020 (0.009 to 0.042)
Total		69	53 076	0.13%	2.5%		
**Ten most likely occupations**							
243. Business, research and administrative professionals	1.2	81	4359	1.86%	2.9%	1.77% (1.36% to 2.18%)	0.19 (0.15 to 0.25)
231. Teaching professionals	2	307	16 057	1.91%	11.1%	1.78% (1.56% to 1.99%)	0.19 (0.17 to 0.23)
352. Legal associate professionals	3	<5	199	1.78%	0.1%	1.92% (0% to 4.14%)	0.21 (0.07 to 0.66)
225. Other health professionals	2	16	616	2.53%	0.6%	1.94% (0.88% to 2.99%)	0.22 (0.13 to 0.38)
117. Health and social services managers and directors	1.1	10	516	1.89%	0.4%	1.98% (0.66% to 3.30%)	0.22 (0.11 to 0.43)
216. Research and development and other research professionals	1.2	5	175	2.63%	0.2%	2.35% (0% to 4.83%)	0.26 (0.09 to 0.73)
241. Legal professionals	1.2	55	2037	2.70%	2.0%	2.46% (1.73% to 3.19%)	0.27 (0.20 to 0.37)
221. Health professionals (excluding doctors)	1.2	46	1558	2.97%	1.7%	2.91% (2.07% to 3.76%)	0.31 (0.23 to 0.43)
211. Natural and social science professionals	1.2	64	2105	3.06%	2.3%	3.09% (2.30% to 3.88%)	0.34 (0.26 to 0.45)
Medical practitioner (doctor)	1.2	453	3292	13.75%	16.3%	10.29% (9.28% to 11.31%)	1 (ref.)
Total		1040	30 915	3.36%	37.5%		

Mean predicted probabilities and risk ratios from multivariable Poisson regression model estimating the association between main earner occupation and being a doctor, adjusting for year turned 18 years old, sex, country of birth, ethnic group and year of survey. The most common NS-SEC analytical class (from 9-class version) for each occupation is also shown for reference. Weighted counts of less than 5 are masked.

NS-SEC, National Statistics Socio-economic Classification; SOC2020, Standard Occupational Classification 2020 version.

Respondents whose main earner during adolescence had routine and semiroutine occupations such as cleaners, home carers, security guards, fork-lift truck, taxi or bus drivers, and warehouse workers, were among the least likely to be doctors, with adjusted mean predicted probabilities of only around 1 in 500 to 1 in 1500 (probability=0.07%–0.18%). These groups were around 50–100 times less likely to be doctors than those whose main earner while growing up was themselves a doctor, for whom the probability was around 1 in 10 (probability=10.3% in model testing association with occupational group, and 10.1% in model testing association with occupational class NS-SEC). Doctor households were far more likely than any other specific occupation group to produce doctors (RR=15.0, 95% CI 13.4 to 16.7, vs all other occupations combined), with the next most likely occupation groups, such as scientists, lawyers, business professionals, teachers, and other health professionals such as speech and language therapists and psychologists, being three to five times less likely.

### Models stratified by year turned 18 years old

**Figure 5:**
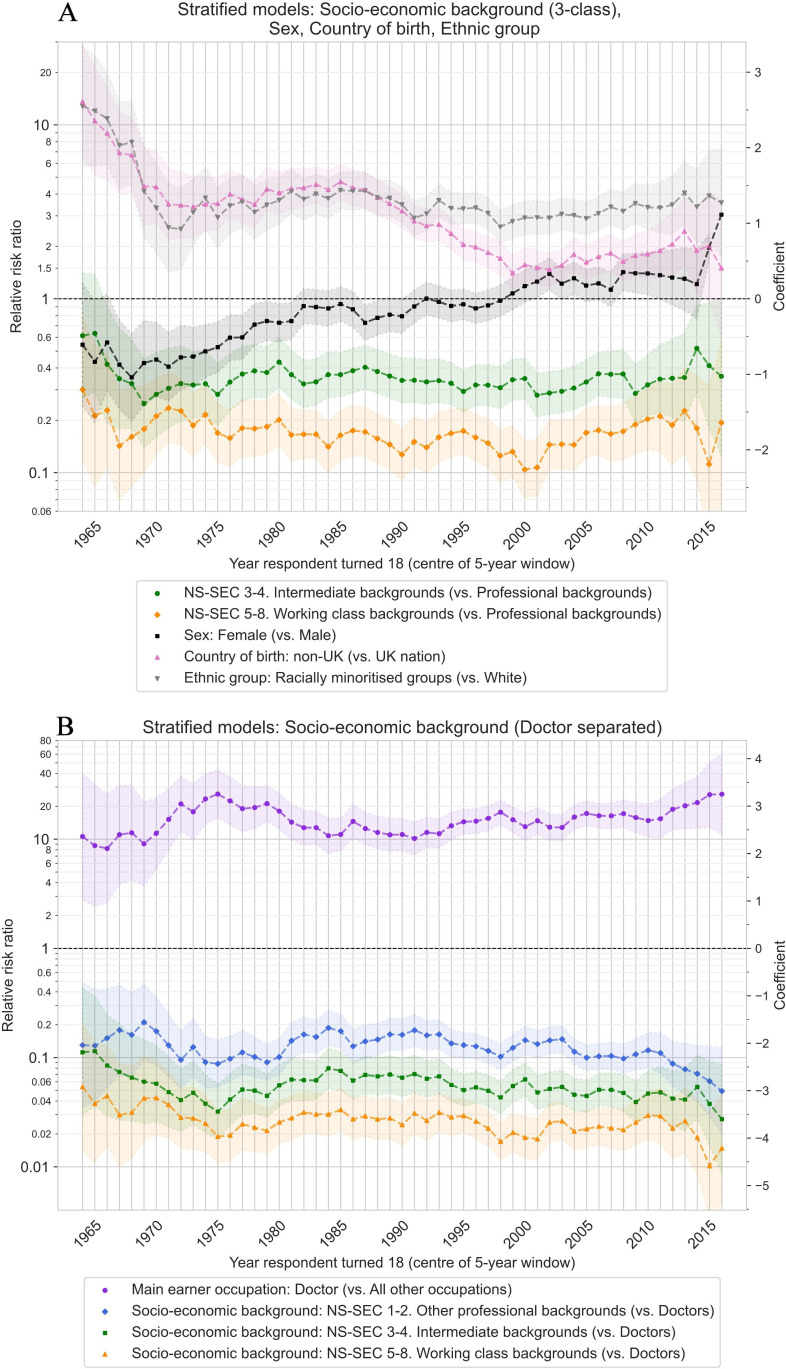
Relative risk ratio with 95% CIs (shaded areas) from multivariable Poisson regression models estimating the associations between sex, country of birth, ethnic group and socioeconomic background, and currently working as a doctor, stratified by year respondent turned 18 years old, using a 5-year rolling window. Models testing the association with: sex adjusted for year of survey; country of birth adjusted for year of survey and sex; ethnic group adjusted for year of survey and sex; socioeconomic background/main earner occupation adjusted for year of survey, sex, country of birth, ethnic group. (**A**) Socioeconomic background modelled as 3-class variable, with NS-SEC 1–2 professional backgrounds as the reference category. (**B**) Socioeconomic background modelled as 3-class variable with a separate category if the main earner was a doctor, with main earner occupation of doctor as the reference category, and effect of doctor as the main earner occupation versus all other occupations. NS-SEC, National Statistics Socio-economic Classification.

Models stratified by year the respondent turned 18 years old as a 5-year rolling window estimated how the likelihood of becoming a doctor for groups with different socioeconomic backgrounds varied over time (figure 5).

Modelling socioeconomic background using the 3-class NS-SEC variable, without separating respondents from doctor backgrounds, the reduced likelihood of intermediate background and working class background respondents being doctors in comparison to professional background respondents remained stable over a time period of 50 years from 1964 to 2016, showing small fluctuations only and no significant trends (figure 5A). In models where those from doctor households were a separate category and acted as the comparison group (figure 5B), small fluctuations were again observed over time, with a weak trend towards widening inequalities between those with and without doctor backgrounds for respondents who turned 18 years old in the most recent years of available data from 2010 to 2016.

In comparison to socioeconomic background, which remained mostly static in terms of social mobility into medicine, changes were observed in the relative representation of doctors by sex, country of birth and ethnic group over time. The likelihood of female sex respondents being doctors steadily increased in cohorts born in later years, switching from being less likely than male counterparts to be doctors to being more likely among those turning 18 years old in the late 1990s onwards. The higher likelihood of respondents born outside of the UK to be doctors in comparison to those born in the UK fell twice, once in the 1960s, and again between 1985 and 2000. From 2000 onwards, respondents born outside of the UK remained 1.5-fold to 2-fold more likely to be doctors. Similarly, the higher likelihood of respondents from racially minoritised ethnic groups (Asian/Asian British, Black/Black British, Mixed/Multiple ethnic groups, and any other ethnic groups) to be doctors in comparison to White ethnic group respondents showed an initial fall in the 1960s, then remained threefold to fourfold more likely from 1970 onwards (in models not controlling for country of birth). Results for disaggregated country of birth and ethnic group variables are given in online supplemental figure S2.

## Discussion

### Key findings

We found large inequalities in the likelihood of working as a doctor in the UK based on socioeconomic background, with those from professional backgrounds three times more likely to become doctors than those with intermediate backgrounds (average predicted probability: 1.6% vs 0.5%), and six times more likely than those with working class backgrounds (average predicted probability: 0.3%) (figure 3 and table 3). The size of the inequality closely follows the average income of the different occupational classes, with higher probabilities for higher income classes.^56^ Despite large shifts in occupational patterns over time and efforts to widen participation in medicine, socioeconomic inequalities remained remarkably consistent and unchanged when comparing those who turned 18 years old (the most common age for starting medical school) at different timepoints from the early 1960s up to the mid-2010s (figure 5). In contrast, trends were observed by sex, country of birth and ethnic group over the same period. Respondents growing up in households where the main earner was a doctor were by far the most likely to themselves become a doctor (average predicted probability: 10.1%), being 15 times more likely to be doctors than respondents with non-doctor backgrounds (RR=15.0, 95% CI 13.4 to 16.7), and between 3 times and 100 times more likely when comparing to other specific occupation groups (figure 4, table 4 and online supplemental table S1).

### Interpretation and strengths

Doctors from professional backgrounds are highly over-represented within medicine, in particular those coming from households where the main earner was also a doctor, resulting in a workforce of doctors that is highly unrepresentative of the general population in terms of socioeconomic background and occupation. This inequality mirrors previous observations within medical school populations over the last 60 years in the UK^2 3 5 7^ and internationally.^10^,^1719 20^ In the closest possible international like-for-like comparison, we found a slightly lower proportion of current doctors from working-class backgrounds in the UK versus USA—14% vs 20% (noting the US figure is based on a much smaller sample of 121 doctors whose socioeconomic background was examined as part of a larger analysis of class pay differences in the 2010s).^57^ However, unlike other studies to date, this analysis is the first to show the persistence and rigidity of these socioeconomic inequalities among working doctors over a time span of 50 years within a single, nationally representative sample. The stability of this inequality over time up to the mid-2010s is consistent with studies finding that recent widening participation schemes did not lead to significant changes in admission profiles in the decade from 2007 to 2016.^7 25 27^ Social mobility data collected in the LFS allowed more detailed comparison than previous studies of specific occupation groups, highlighting the 50-fold to 100-fold lower probability of becoming a doctor for those coming from households where the main earner worked in routine and semiroutine occupations such as cleaners, home carers, security guards, forklift truck, taxi or bus drivers, and warehouse workers, compared with those from doctor households (table 4). While we highlight doctors coming from doctor backgrounds as an extreme outlier, we emphasise that this outlier group was not solely responsible for the observed socioeconomic inequalities, with evidence of large differences in the likelihood of being a doctor observed between other professional occupations and working-class occupations that are, on average, less well paid, associated with lower social, cultural and economic capital,^58^ and concentrated in more marginalised and deprived areas.^59^ However, the extent to which doctor households remain an outlier among more privileged backgrounds is also noteworthy, with respondents from doctor households still eightfold more likely to become doctors than those from non-doctor professional backgrounds (table 3, model 3), and at least threefold more likely than any other detailed occupation group (figure 4 and table 4). In the wider context of occupational reproduction across generations, doctors have been found to have the highest level of so-called ‘microclass reproduction’ in analysis of workers in 2010s’ UK.^34^ Offspring of doctors were much more likely to work in the same occupation as their parents/carers than other high-income professions such as law, architecture or finance. Similarly, social mobility analysis of data from 1962 to 2002 in Germany, Japan, Sweden and the USA found doctors had the highest microclass reproduction after airline pilots, ship officers and clergy.^60^

### Implications for clinicians and policymakers

Is the lack of representation among doctors of people from less affluent backgrounds a problem? We hypothesise that the poorer care experiences reported by less privileged patients^35^,^39^ may be in part due to socioeconomic imbalances or ‘discordance’ between patients and doctors, akin to previous reports showing race concordance between patient and doctor was associated with better communication and experiences among Black Americans.^43 61 62^ While the need to be open to those from all backgrounds and parts of society is an integral part of medical education, it is conceivable that doctors who are from more affluent backgrounds than their patients may be less relatable, and less likely to have lived experience and awareness of the wider difficulties that patients face compared with those from less affluent backgrounds. More affluent doctors may be more susceptible to the implicit bias against less affluent patients that has been previously observed.^42^ This is particularly pertinent given the growing focus on holistic care that takes into account social and economic circumstances that impact health,^63^ echoing earlier reports by less privileged groups on what makes a good GP consultation, who highlighted continuity of care, easy-to-understand communication, genuine empathy, relatability, and understanding the ‘bigger picture’ of patients’ socioeconomic contexts.^64 65^ If this hypothesis is true, increasing the number of less privileged doctors as well as giving further training to current doctors may lead to improved care. Beyond patient-doctor interactions, such imbalances within the medical profession may also contribute to structural forms of inequity and systemic inattention to pressing issues, such as primary care resource constraints in more deprived areas,^66^ or limited reflection on socioeconomic inequalities in increasingly digital care.^67^ A greater understanding of such inequities through closer experience may aid their recognition and responses in policy debate.

To fully understand the extent to which socioeconomic differences between doctors and patients affect care experiences and health system policy, we first require comprehensive data on doctors’ socioeconomic backgrounds to be collected and made available for research. This has not happened to date in the UK, despite being highlighted as a priority in a 2012 UK Government report,^68^ and guidance on how to do so being published by the UK Government’s Social Mobility Commission.^49^ Collection could be incorporated into the national databases by the UK medical register or NHS England both of which already record characteristics protected in the UK Equality Act. Similar calls for improved data collection have also been made in the Netherlands to address the ‘leaky pipeline’ of declining diversity during medical training.^11^ How can such inequities be addressed? The root causes are complex and likely to have changed over time, requiring multifaceted efforts. Socioeconomic inequalities along the pipeline to becoming and remaining a doctor need to be addressed, but also perhaps more crucially, structural and societal barriers that prevent capable students from applying to medical school, in the first place, need to be dismantled. Exposure to role models and addressing cultural perceptions, attitudes and stigma that act against lower socioeconomic students and make them feel like medicine ‘isn’t for people like them’^669^,^72^ could be a key focus within educational settings throughout childhood, demonstrating that traditionally high-status careers such as medicine are legitimate, feasible and rewarding options for all. But policy is needed to ensure this is true—government and higher education institutions could also work to address financial and geographical barriers to application,^70 73 74^ provide additional support to disadvantaged students during studies to compensate for lack of ‘insider knowledge’ of the ‘hidden curriculum’ from which those from medical or professional families benefit,^75 76^ and equalise the skewed geography of widening participation initiatives.^77^ Meanwhile, addressing declining pay for junior doctors,^78^ as well as working conditions and declining job enthusiasm among current doctors,^79^ is also likely to increase the appeal of medicine and reduce the pressure to stop studies,^80^ especially among less affluent students. However, given the apparent stubbornness of inequalities in access to medicine, it may be more useful to shift focus to understanding and addressing any repercussions for patients of socioeconomic inequality among doctors. This may include studying whether socioeconomic concordance in patient-doctor dyads affects patient-rated experiences, and/or developing ‘cultural competence’ training of practising doctors to share learnings from previous studies detailing the needs of lower socioeconomic status patients in patient-doctor interactions.^64 65^

### Limitations

While our sample size is large and designed to be age-representative and sex-representative at a regional level, the number of doctors in our sample (2772) remains a small proportion (≈1%–2%) of the total workforce and so results may not be generalisable to the whole workforce of doctors—there were 45 000 doctors in general practice and 134 000 full-time equivalent doctors in hospital and community health services in NHS in England in July 2023.^81 82^ We note that 24% of respondents in the desired age range and 22% of current doctors were excluded due to data on socioeconomic background being missing, which we attempted to adjust for with inverse probability weighting. We focused on socioeconomic background as our main exposure due to the current absence of comprehensive data collection, but acknowledge the importance of understanding how inequalities vary for different intersections of socioeconomic background and other factors such as sex/gender and race/ethnicity, which should be expanded on in future with further intersectional analysis. Furthermore, given the correlation between race/ethnicity and socioeconomic status, resulting from complex histories of structural discrimination in many countries across the world, it will be important to understand whether intersectional inequalities vary across countries, for example between the UK and USA. Finally, we note that the NS-SEC measure of occupational class is a single indicator of socioeconomic status, and that collection of further indicators of socioeconomic status during childhood, as outlined by Social Mobility Commission guidance,^49^ or those used in other countries (eg, the parental education occupation indicator used by US medical schools^83^), could provide a more detailed understanding of inequalities among both medical students and current doctors. Because the LFS focuses on current employment, we focus on inequalities among current doctors and are unable to look in detail at the most recent trends in entrants to medical schools, with data covering those who likely entered medical school up to the mid-2010s only. Other more complete data sets of university applications and entrants are better suited to such analyses and have found similar socioeconomic inequalities.^9^

### Summary

We found large inequalities in the socioeconomic backgrounds of doctors in the UK that were persistent over time, leading to poor representativeness of doctors of the general population at the national level. New data collections on socioeconomic background within the British medical profession are required if we are to understand the effects of poor socioeconomic representation among doctors on patient care, as well as better track widening participation initiatives.

## Supplementary material

10.1136/bmjopen-2024-097178 online supplemental file 1

## Data Availability

Data may be obtained from a third party and are not publicly available.
