# Supplementary material for: Socioeconomic diversity of doctors in the United Kingdom: a cross-sectional study of 10 years of Labour Force Survey social mobility data
Source: BMJ Open. 2025 Sep 9;15(9):e097178. doi: 10.1136/bmjopen-2024-097178 (PMC12421607; doi:10.1136/bmjopen-2024-097178)
Supplement: online supplemental file 1 [file bmjopen-15-9-s001.docx]

# Supporting information

# Socio-economic diversity of Doctors in the United Kingdom: A cross-sectional study of 10 years of Labour Force Survey social mobility data

## Author list

Nathan J Cheetham^1^*, Fleur Cantle^2^, Andy Guise^3^, Claire J Steves^1,4^*

* Corresponding authors

Correspondence to: [nathan.cheetham@kcl.ac.uk](mailto:nathan.cheetham@kcl.ac.uk), [claire.j.steves@kcl.ac.uk](mailto:claire.j.steves@kcl.ac.uk)

1 Department of Twin Research and Genetic Epidemiology, King’s College London, London, United Kingdom

2 Department of Emergency Medicine, King's College Hospital, London, United Kingdom

3 Department of Population Health Sciences, King’s College London, London, United Kingdom

4 Guy’s & St Thomas’s NHS Foundation Trust, London, United Kingdom

## Association between socio-demographics and being a doctor


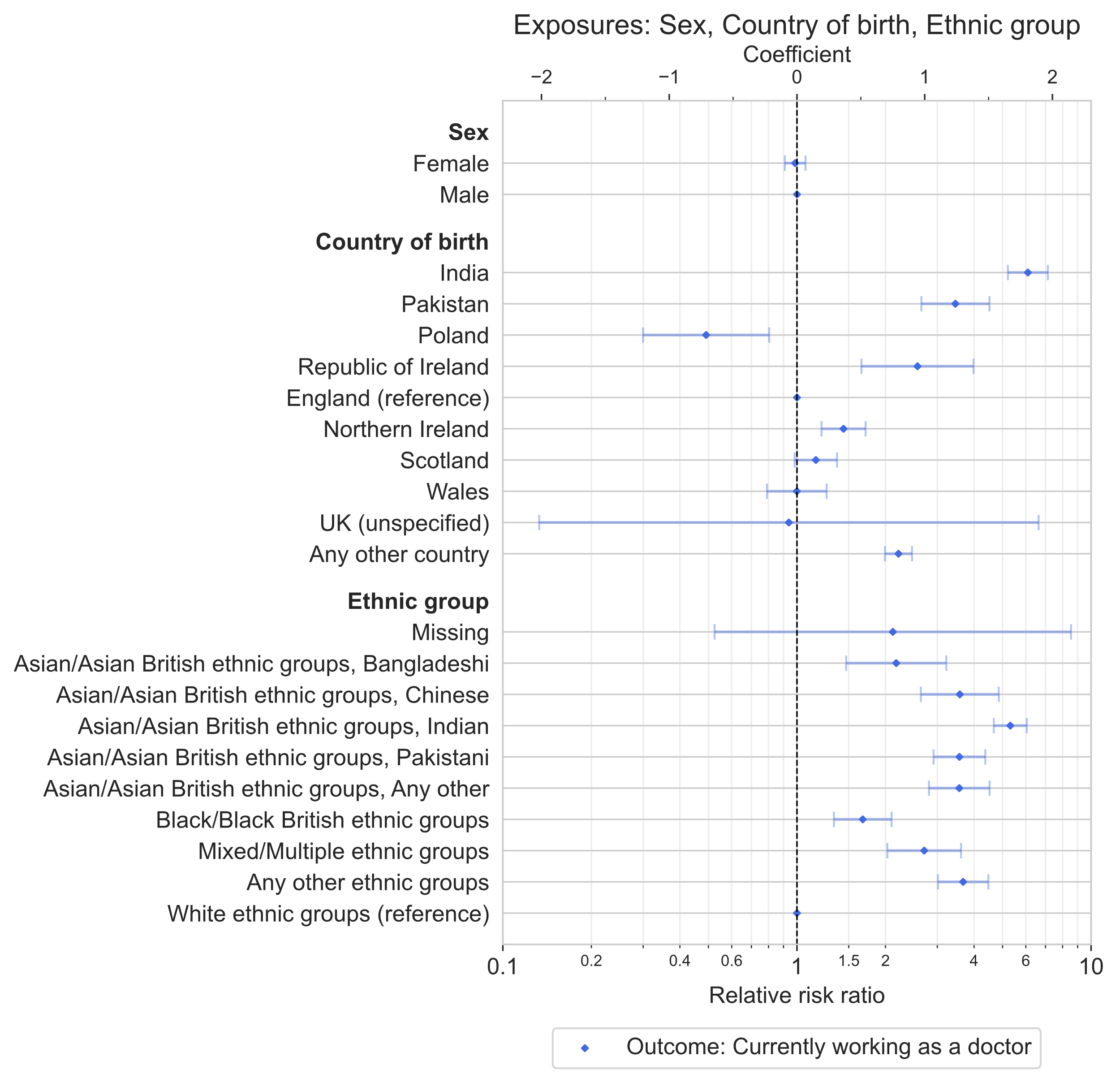


Figure S 1. Relative risk ratios from poisson regression models estimating the association between sex, country of birth, and ethnic group and likelihood of working as a doctor. Models estimating association with: sex adjusted for year respondent turned 18 and year of survey; country of birth adjusted for year respondent turned 18, year of survey and sex; ethnic group adjusted for year respondent turned 18, year of survey and sex.

## DAG code (for reproduction with daggity software)

dag {

"Country of birth" [pos="-0.473,-0.572"]

"Currently working as a doctor" [outcome,pos="-0.465,-0.543"]

"Ethnic group" [pos="-0.473,-0.563"]

"Main earner NSSEC" [pos="-0.350,-0.553"]

"Main earner occupation" [pos="-0.569,-0.553"]

"Year of survey" [pos="-0.472,-0.591"]

"Year respondent turned 18" [pos="-0.590,-0.581"]

Sex [pos="-0.363,-0.581"]

"Country of birth" -> "Ethnic group"

"Ethnic group" -> "Main earner occupation"

"Main earner NSSEC" -> "Currently working as a doctor"

"Main earner occupation" -> "Main earner NSSEC"

"Year of survey" -> "Year respondent turned 18"

"Year of survey" -> Sex

"Year respondent turned 18" -> "Country of birth"

"Year respondent turned 18" <-> Sex

Sex -> "Country of birth"

}

## Stratified models testing association between disaggregated socio-demographics and working as a doctor


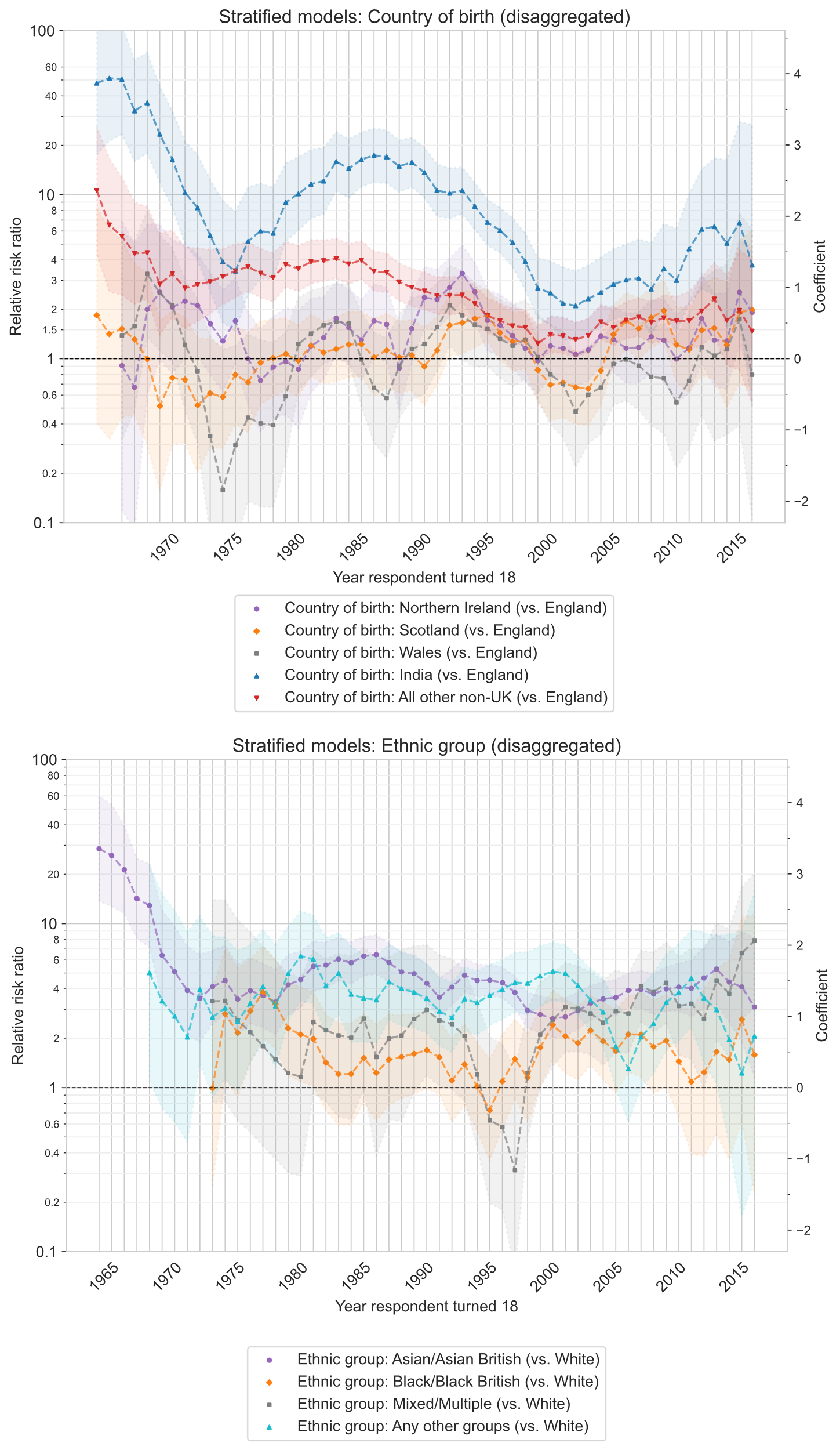


Figure S 2. Relative risk ratio with 95% confidence intervals (shaded areas) from multivariable poisson regression models estimating the association between country of birth (top) and ethnic group (bottom) and currently working as a doctor, stratified by year respondent turned 18 using a 5-year rolling window. Models testing the association with: country of birth adjusted for year of survey and sex; ethnic group adjusted for year of survey and sex.

## Full results tables

### Association between main earner occupation and working as a doctor

Table S 1. Summary of unadjusted weighted counts and adjusted results from multivariable poisson regression model estimating the association between main earner occupation at age 14 and likelihood of working as a doctor. Multivariable models adjusted for year respondent turned 18, sex, country of birth, ethnic group and year of survey. Most common NS-SEC categories (3-class and 9-class versions) for each occupation group are also given for reference. 9 of 106 occupation groups with either no current doctors or less than 100 respondents are not presented. Weighted counts of less than 5 are masked.

|  |  |  | **Unadjusted counts (weighted)** | | | | **Adjusted mean predicted probability** | | | **Adjusted Risk Ratio** | | |
| --- | --- | --- | --- | --- | --- | --- | --- | --- | --- | --- | --- | --- |
| **SOC2020 Occupation code** | **NS-SEC (9-class)** | **NS-SEC (3-class)** | **Current doctors** | **Respondents** | **% respondents** | **% doctors** | **Probability (%)** | **95% CI lower** | **95% CI upper** | **Risk Ratio** | **95% CI lower** | **95% CI upper** |
| Medical practitioners (Doctors) | 1.2 | 1-2. Professional background | 453 | 3292 | 13.75% | 16.3% | 10.29% | 9.28% | 11.31% | 1 (reference) |  |  |
| 211. Natural and social science professionals | 1.2 | 1-2. Professional background | 64 | 2105 | 3.06% | 2.3% | 3.09% | 2.30% | 3.88% | 0.340 | 0.259 | 0.446 |
| 221. Health professionals (excluding Doctors) | 1.2 | 1-2. Professional background | 46 | 1558 | 2.97% | 1.7% | 2.91% | 2.07% | 3.76% | 0.315 | 0.232 | 0.428 |
| 241. Legal professionals | 1.2 | 1-2. Professional background | 55 | 2037 | 2.70% | 2.0% | 2.46% | 1.73% | 3.19% | 0.270 | 0.198 | 0.368 |
| 216. Research and development (r&d) and other research professionals | 1.2 | 1-2. Professional background | 5 | 175 | 2.63% | 0.2% | 2.35% | 0.00% | 4.83% | 0.260 | 0.093 | 0.729 |
| 117. Health and social services managers and directors | 1.1 | 1-2. Professional background | 10 | 516 | 1.89% | 0.4% | 1.98% | 0.66% | 3.30% | 0.218 | 0.111 | 0.427 |
| 225. Other health professionals | 2 | 1-2. Professional background | 16 | 616 | 2.53% | 0.6% | 1.94% | 0.88% | 2.99% | 0.218 | 0.126 | 0.379 |
| 352. Legal associate professionals | 3 | 3-4. Intermediate background | <5 | 199 | 1.78% | 0.1% | 1.92% | 0.00% | 4.14% | 0.211 | 0.067 | 0.659 |
| 231. Teaching professionals | 2 | 1-2. Professional background | 307 | 16057 | 1.91% | 11.1% | 1.78% | 1.56% | 1.99% | 0.195 | 0.167 | 0.227 |
| 243. Business, research and administrative professionals | 1.2 | 1-2. Professional background | 81 | 4359 | 1.86% | 2.9% | 1.77% | 1.36% | 2.18% | 0.192 | 0.149 | 0.247 |
| 111. Chief executives and senior officials | 1.1 | 1-2. Professional background | 30 | 1470 | 2.02% | 1.1% | 1.74% | 1.04% | 2.44% | 0.190 | 0.126 | 0.287 |
| 354. Business associate professionals | 2 | 1-2. Professional background | 8 | 388 | 2.01% | 0.3% | 1.58% | 0.16% | 3.00% | 0.178 | 0.072 | 0.438 |
| 246. Welfare professionals | 2 | 1-2. Professional background | 31 | 2134 | 1.43% | 1.1% | 1.46% | 0.93% | 2.00% | 0.161 | 0.110 | 0.235 |
| 245. Architects, chartered architectural technologists, planning officers, surveyors and construction professionals | 1.2 | 1-2. Professional background | 46 | 3516 | 1.30% | 1.6% | 1.44% | 1.00% | 1.88% | 0.159 | 0.115 | 0.220 |
| 212. Engineering professionals | 1.2 | 1-2. Professional background | 136 | 10233 | 1.33% | 4.9% | 1.42% | 1.17% | 1.67% | 0.156 | 0.127 | 0.190 |
| 249. Media professionals | 2 | 1-2. Professional background | 10 | 736 | 1.33% | 0.4% | 1.41% | 0.54% | 2.29% | 0.155 | 0.083 | 0.291 |
| 113. Functional managers and directors | 1.1 | 1-2. Professional background | 115 | 9177 | 1.25% | 4.1% | 1.23% | 0.98% | 1.48% | 0.135 | 0.108 | 0.169 |
| 412. Administrative occupations: finance | 3 | 3-4. Intermediate background | 74 | 5897 | 1.26% | 2.7% | 1.16% | 0.85% | 1.46% | 0.128 | 0.096 | 0.169 |
| 112. Production managers and directors | 1.1 | 1-2. Professional background | 86 | 7658 | 1.12% | 3.1% | 1.15% | 0.88% | 1.42% | 0.126 | 0.098 | 0.163 |
| 232. Other educational professionals | 1.2 | 1-2. Professional background | 8 | 636 | 1.30% | 0.3% | 1.15% | 0.32% | 1.97% | 0.130 | 0.063 | 0.268 |
| 343. Sports and fitness occupations | 2 | 1-2. Professional background | <5 | 302 | 1.08% | 0.1% | 1.14% | 0.00% | 2.45% | 0.126 | 0.040 | 0.395 |
| 341. Artistic, literary and media occupations | 2 | 1-2. Professional background | 20 | 1819 | 1.08% | 0.7% | 1.10% | 0.55% | 1.64% | 0.121 | 0.073 | 0.201 |
| 313. Information technology technicians | 2 | 1-2. Professional background | 6 | 548 | 1.08% | 0.2% | 1.09% | 0.11% | 2.08% | 0.121 | 0.049 | 0.299 |
| 358. Regulatory associate professionals | 2 | 1-2. Professional background | <5 | 348 | 1.13% | 0.1% | 1.08% | 0.00% | 2.39% | 0.122 | 0.037 | 0.411 |
| 247. Librarians and related professionals | 2 | 1-2. Professional background | <5 | 344 | 1.03% | 0.1% | 1.08% | 0.01% | 2.14% | 0.119 | 0.044 | 0.319 |
| 414. Administrative occupations: office managers and supervisors | 2 | 1-2. Professional background | 23 | 1791 | 1.29% | 0.8% | 1.07% | 0.56% | 1.59% | 0.117 | 0.071 | 0.190 |
| 411. Administrative occupations: government and related organisations | 3 | 3-4. Intermediate background | 64 | 5135 | 1.25% | 2.3% | 1.07% | 0.79% | 1.35% | 0.117 | 0.088 | 0.155 |
| 222. Therapy professionals | 2 | 1-2. Professional background | 5 | 467 | 1.08% | 0.2% | 1.06% | 0.10% | 2.02% | 0.118 | 0.047 | 0.292 |
| 213. Information technology professionals | 2 | 1-2. Professional background | 37 | 3582 | 1.02% | 1.3% | 1.05% | 0.68% | 1.42% | 0.116 | 0.080 | 0.167 |
| 351. Transport associate professionals | 2 | 1-2. Professional background | 11 | 1172 | 0.94% | 0.4% | 1.02% | 0.38% | 1.66% | 0.112 | 0.060 | 0.212 |
| 244. Business and financial project management professionals | 1.2 | 1-2. Professional background | <5 | 230 | 1.11% | 0.1% | 1.00% | 0.00% | 2.68% | 0.113 | 0.022 | 0.593 |
| 321. Health associate professionals | 3 | 3-4. Intermediate background | 5 | 578 | 0.86% | 0.2% | 0.94% | 0.00% | 1.92% | 0.103 | 0.036 | 0.295 |
| 415. Other administrative occupations | 3 | 3-4. Intermediate background | 29 | 2971 | 0.98% | 1.1% | 0.94% | 0.56% | 1.31% | 0.103 | 0.068 | 0.156 |
| 611. Teaching and childcare support occupations | 3 | 3-4. Intermediate background | 15 | 1691 | 0.91% | 0.6% | 0.92% | 0.37% | 1.48% | 0.102 | 0.055 | 0.188 |
| 353. Finance associate professionals | 2 | 1-2. Professional background | 33 | 3776 | 0.86% | 1.2% | 0.88% | 0.54% | 1.21% | 0.095 | 0.064 | 0.142 |
| 242. Finance professionals | 1.2 | 1-2. Professional background | 11 | 1115 | 1.00% | 0.4% | 0.86% | 0.22% | 1.51% | 0.098 | 0.046 | 0.207 |
| 322. Welfare and housing associate professionals | 2 | 1-2. Professional background | 8 | 968 | 0.84% | 0.3% | 0.86% | 0.11% | 1.60% | 0.094 | 0.039 | 0.226 |
| 612. Animal care and control services | 7 | 5-8. Working class background | <5 | 233 | 0.81% | 0.1% | 0.85% | 0.00% | 2.52% | 0.093 | 0.013 | 0.638 |
| 121. Managers and proprietors in agriculture related services | 4 | 3-4. Intermediate background | 6 | 762 | 0.85% | 0.2% | 0.82% | 0.20% | 1.45% | 0.090 | 0.042 | 0.193 |
| 123. Managers and proprietors in health and care services | 2 | 1-2. Professional background | 6 | 729 | 0.80% | 0.2% | 0.82% | 0.15% | 1.50% | 0.091 | 0.040 | 0.207 |
| 311. Science, engineering and production technicians | 2 | 1-2. Professional background | 18 | 2427 | 0.74% | 0.6% | 0.80% | 0.41% | 1.20% | 0.088 | 0.053 | 0.146 |
| 342. Design occupations | 4 | 3-4. Intermediate background | <5 | 614 | 0.73% | 0.2% | 0.80% | 0.00% | 1.69% | 0.088 | 0.029 | 0.266 |
| 355. Sales, marketing and related associate professionals | 2 | 1-2. Professional background | 45 | 6057 | 0.75% | 1.6% | 0.78% | 0.54% | 1.03% | 0.086 | 0.062 | 0.120 |
| 116. Senior officers in protective services | 1.1 | 1-2. Professional background | 27 | 3217 | 0.85% | 1.0% | 0.75% | 0.44% | 1.07% | 0.082 | 0.054 | 0.126 |
| 115. Managers and directors in retail and wholesale | 2 | 1-2. Professional background | 19 | 2850 | 0.68% | 0.7% | 0.70% | 0.38% | 1.02% | 0.077 | 0.048 | 0.123 |
| 624. Cleaning and housekeeping managers and supervisors | 5 | 5-8. Working class background | <5 | 331 | 0.69% | 0.1% | 0.68% | 0.00% | 1.65% | 0.075 | 0.019 | 0.305 |
| 125. Managers and proprietors in other services | 4 | 3-4. Intermediate background | 76 | 9304 | 0.81% | 2.7% | 0.67% | 0.51% | 0.84% | 0.073 | 0.056 | 0.095 |
| 812. Metal working machine operatives | 6 | 5-8. Working class background | 6 | 778 | 0.83% | 0.2% | 0.67% | 0.21% | 1.13% | 0.076 | 0.038 | 0.152 |
| 312. Cad, drawing and architectural technicians | 3 | 3-4. Intermediate background | 7 | 1334 | 0.53% | 0.3% | 0.67% | 0.22% | 1.11% | 0.073 | 0.037 | 0.145 |
| 356. Public services associate professionals | 2 | 1-2. Professional background | <5 | 246 | 0.70% | 0.1% | 0.63% | 0.00% | 1.52% | 0.071 | 0.017 | 0.296 |
| 223. Nursing professionals | 2 | 1-2. Professional background | 32 | 5117 | 0.62% | 1.2% | 0.62% | 0.38% | 0.86% | 0.068 | 0.046 | 0.102 |
| 122. Managers and proprietors in hospitality and leisure services | 4 | 3-4. Intermediate background | 32 | 4652 | 0.69% | 1.2% | 0.60% | 0.38% | 0.82% | 0.066 | 0.045 | 0.097 |
| 533. Construction and building trades supervisors | 5 | 5-8. Working class background | 6 | 1088 | 0.51% | 0.2% | 0.58% | 0.06% | 1.10% | 0.064 | 0.026 | 0.157 |
| 357. HR, training and other vocational associate guidance professionals | 2 | 1-2. Professional background | 12 | 2368 | 0.52% | 0.4% | 0.58% | 0.28% | 0.88% | 0.064 | 0.038 | 0.107 |
| 421. Secretarial and related occupations | 3 | 3-4. Intermediate background | 18 | 3455 | 0.51% | 0.6% | 0.56% | 0.30% | 0.82% | 0.062 | 0.038 | 0.100 |
| 525. Skilled metal, electrical and electronic trades supervisors | 2 | 1-2. Professional background | <5 | 839 | 0.47% | 0.1% | 0.55% | 0.01% | 1.10% | 0.061 | 0.023 | 0.164 |
| 331. Protective service occupations | 3 | 3-4. Intermediate background | 41 | 7736 | 0.52% | 1.5% | 0.53% | 0.35% | 0.72% | 0.059 | 0.041 | 0.084 |
| 524. Electrical and electronic trades | 5 | 5-8. Working class background | 50 | 10638 | 0.47% | 1.8% | 0.50% | 0.36% | 0.65% | 0.056 | 0.041 | 0.076 |
| 413. Administrative occupations: records | 3 | 3-4. Intermediate background | 9 | 1903 | 0.47% | 0.3% | 0.49% | 0.16% | 0.83% | 0.054 | 0.027 | 0.108 |
| 623. Housekeeping and related services | 6 | 5-8. Working class background | 5 | 1330 | 0.40% | 0.2% | 0.46% | 0.06% | 0.87% | 0.051 | 0.021 | 0.124 |
| 248. Quality and regulatory professionals | 2 | 1-2. Professional background | <5 | 715 | 0.43% | 0.1% | 0.45% | 0.00% | 0.97% | 0.050 | 0.016 | 0.159 |
| 542. Printing trades | 5 | 5-8. Working class background | 8 | 2157 | 0.38% | 0.3% | 0.45% | 0.10% | 0.81% | 0.050 | 0.023 | 0.110 |
| 215. Conservation and environment professionals | 1.2 | 1-2. Professional background | <5 | 191 | 0.41% | 0.0% | 0.42% | 0.00% | 1.26% | 0.046 | 0.007 | 0.332 |
| 721. Customer service occupations | 3 | 5-8. Working class background | <5 | 1047 | 0.38% | 0.1% | 0.38% | 0.02% | 0.74% | 0.042 | 0.016 | 0.108 |
| 124. Managers in logistics, warehousing and transport | 2 | 1-2. Professional background | 10 | 2714 | 0.36% | 0.3% | 0.38% | 0.13% | 0.63% | 0.042 | 0.021 | 0.081 |
| 712. Sales related occupations | 3 | 3-4. Intermediate background | 16 | 4334 | 0.38% | 0.6% | 0.38% | 0.17% | 0.58% | 0.041 | 0.024 | 0.071 |
| 511. Agricultural and related trades | 4 | 3-4. Intermediate background | 54 | 11783 | 0.46% | 1.9% | 0.37% | 0.27% | 0.47% | 0.040 | 0.030 | 0.054 |
| 921. Elementary administration occupations | 6 | 5-8. Working class background | 11 | 3168 | 0.33% | 0.4% | 0.36% | 0.09% | 0.63% | 0.040 | 0.019 | 0.085 |
| 713. Shopkeepers and sales supervisors | 4 | 3-4. Intermediate background | 13 | 2597 | 0.50% | 0.5% | 0.36% | 0.12% | 0.60% | 0.040 | 0.021 | 0.079 |
| 523. Vehicle trades | 5 | 5-8. Working class background | 22 | 6818 | 0.32% | 0.8% | 0.35% | 0.20% | 0.50% | 0.039 | 0.025 | 0.060 |
| 531. Construction and building trades | 4 | 3-4. Intermediate background | 60 | 19728 | 0.30% | 2.2% | 0.34% | 0.25% | 0.42% | 0.037 | 0.028 | 0.049 |
| 522. Metal machining, fitting and instrument making trades | 5 | 5-8. Working class background | 25 | 8893 | 0.28% | 0.9% | 0.33% | 0.19% | 0.46% | 0.036 | 0.024 | 0.055 |
| 814. Assemblers and routine operatives | 6 | 5-8. Working class background | 12 | 3624 | 0.32% | 0.4% | 0.32% | 0.12% | 0.52% | 0.035 | 0.019 | 0.066 |
| 544. Other skilled trades | 7 | 5-8. Working class background | 7 | 2487 | 0.30% | 0.3% | 0.31% | 0.07% | 0.56% | 0.035 | 0.016 | 0.076 |
| 622. Hairdressers and related services | 7 | 5-8. Working class background | <5 | 924 | 0.31% | 0.1% | 0.30% | 0.00% | 0.72% | 0.033 | 0.008 | 0.134 |
| 815. Construction operatives | 6 | 5-8. Working class background | 6 | 2449 | 0.26% | 0.2% | 0.30% | 0.06% | 0.54% | 0.033 | 0.015 | 0.074 |
| Does not apply | 8 | 5-8. Working class background | 58 | 20478 | 0.28% | 2.1% | 0.27% | 0.19% | 0.35% | 0.030 | 0.022 | 0.040 |
| 823. Other drivers and transport operatives | 5 | 5-8. Working class background | 7 | 2998 | 0.24% | 0.3% | 0.26% | 0.05% | 0.48% | 0.029 | 0.013 | 0.066 |
| 521. Metal forming, welding and related trades | 7 | 5-8. Working class background | 8 | 3852 | 0.21% | 0.3% | 0.25% | 0.10% | 0.41% | 0.028 | 0.015 | 0.051 |
| 816. Production, factory and assembly supervisors | 5 | 5-8. Working class background | <5 | 275 | 0.30% | 0.0% | 0.25% | 0.00% | 0.75% | 0.029 | 0.004 | 0.204 |
| 912. Elementary construction occupations | 7 | 5-8. Working class background | 9 | 4125 | 0.22% | 0.3% | 0.23% | 0.05% | 0.41% | 0.025 | 0.011 | 0.057 |
| 541. Textiles and garments trades | 7 | 5-8. Working class background | 6 | 2331 | 0.27% | 0.2% | 0.23% | 0.04% | 0.42% | 0.025 | 0.011 | 0.058 |
| 811. Process operatives | 6 | 5-8. Working class background | 18 | 7897 | 0.23% | 0.6% | 0.23% | 0.12% | 0.33% | 0.025 | 0.015 | 0.040 |
| 911. Elementary agricultural occupations | 6 | 5-8. Working class background | 6 | 3004 | 0.21% | 0.2% | 0.21% | 0.04% | 0.38% | 0.023 | 0.010 | 0.051 |
| 913. Elementary process plant occupations | 7 | 5-8. Working class background | 18 | 8563 | 0.21% | 0.6% | 0.21% | 0.11% | 0.31% | 0.023 | 0.014 | 0.038 |
| 711. Sales assistants and retail cashiers | 6 | 5-8. Working class background | 11 | 5449 | 0.21% | 0.4% | 0.20% | 0.07% | 0.33% | 0.022 | 0.012 | 0.042 |
| 543. Food preparation and hospitality trades | 5 | 5-8. Working class background | 11 | 5086 | 0.22% | 0.4% | 0.20% | 0.07% | 0.32% | 0.021 | 0.011 | 0.041 |
| 926. Other elementary services occupations | 7 | 5-8. Working class background | 7 | 3771 | 0.18% | 0.2% | 0.18% | 0.05% | 0.31% | 0.020 | 0.009 | 0.042 |
| 532. Building finishing trades | 4 | 3-4. Intermediate background | 7 | 4595 | 0.14% | 0.2% | 0.18% | 0.02% | 0.33% | 0.019 | 0.008 | 0.046 |
| 922. Elementary cleaning occupations | 7 | 5-8. Working class background | 8 | 5878 | 0.14% | 0.3% | 0.16% | 0.03% | 0.28% | 0.017 | 0.007 | 0.039 |
| 925. Elementary storage occupations | 7 | 5-8. Working class background | 6 | 4289 | 0.13% | 0.2% | 0.15% | 0.04% | 0.27% | 0.017 | 0.008 | 0.036 |
| 821. Road transport drivers | 7 | 5-8. Working class background | 22 | 15502 | 0.14% | 0.8% | 0.15% | 0.08% | 0.22% | 0.016 | 0.010 | 0.026 |
| 621. Leisure and travel services | 6 | 5-8. Working class background | <5 | 1253 | 0.16% | 0.1% | 0.15% | 0.00% | 0.35% | 0.016 | 0.004 | 0.065 |
| 613. Caring personal services | 6 | 5-8. Working class background | 7 | 5381 | 0.12% | 0.2% | 0.13% | 0.03% | 0.23% | 0.014 | 0.007 | 0.030 |
| 813. Plant and machine operatives | 7 | 5-8. Working class background | 8 | 7757 | 0.10% | 0.3% | 0.12% | 0.04% | 0.21% | 0.014 | 0.007 | 0.028 |
| 923. Elementary security occupations | 6 | 5-8. Working class background | <5 | 2374 | 0.07% | 0.1% | 0.08% | 0.00% | 0.19% | 0.008 | 0.002 | 0.035 |
| 822. Mobile machine drivers and operatives | 6 | 5-8. Working class background | <5 | 2275 | 0.06% | 0.1% | 0.07% | 0.00% | 0.17% | 0.008 | 0.002 | 0.032 |
