# Supplementary material for: Socioeconomic diversity of doctors in the United Kingdom: a cross-sectional study of 10 years of Labour Force Survey social mobility data
Source: BMJ Open. 2025 Sep 9;15(9):e097178. doi: 10.1136/bmjopen-2024-097178 (PMC12421607; doi:10.1136/bmjopen-2024-097178)
Supplement: Supplementary file 2 [file bmjopen-2024-097178.reviewer_comments.pdf]

PEER REVIEW HISTORY

BMJ Open publishes all reviews undertaken for accepted manuscripts. Reviewers are asked to complete a checklist review form and are provided with free text boxes to elaborate on their assessment. These free text comments are reproduced below.

ARTICLE DETAILS

Title (Provisional)

Socio-economic diversity of Doctors in the United Kingdom: A cross-sectional study of 10 years of Labour Force Survey social mobility data

Authors

Cheetham, Nathan J.; Cantle, Fleur; Guise, Andy; Steves, Claire J.

VERSION 1 - REVIEW

**Reviewer**	**1**
**Name**	**Shahriar , Arman**
**Affiliation**	**The University of Chicago, Internal Medicine**
**Date**	**28-Dec-2024**
**COI**	**None**

Summary
In this observational study, the researchers assessed the effect of childhood socioeconomic status (SES) – as estimated by the occupation of the main household earner when the respondent was 14 years old – on ultimate occupation as a physician in the UK. The methods are robust. The results are unique. The figures are excellent. The primary finding was a strong association between childhood SES and occupation as a physician. One important secondary finding was the magnitude of this observation among adolescents who hail from "doctor households" (11.5-13% probability; 15-fold more likely than those without physicians who are parents). The results are presented nicely in disaggregated fashion in Table 2 and summarized visually in Figure 2 – the key figure in this study. My comments are minor and stratified by section.

Methods
On page 5, line 49 the authors write:
"The exposures of interest were the occupation and socioeconomic status of the main earner of the household when respondent was aged 14". As far as I can tell, socioeconomic status was *entirely* derived from occupation – which mapped to NS-SEC groups based on the Standard Occupational Classification system presented in Table 1. Thus, using the word "and" in the above sentence may be misleading. With that said, parental occupation has been validated as a proxy for SES in American literature (Grbic D et al *Acad Med* 2015; 90(7): 953-960).

The missingness percentage (24%) is somewhat concerning, but most of us know from this sort of survey data, expected. Given the 24% missing data for a survey, I would recommend an assessment by a statistical reviewer (if other peer reviewers are not comfortable providing an assessment). They do discuss this in limitations (page 22)

Results
The authors do a fantastic job of summarizing findings using appropriate visuals that can leave less scientific or even non-scientific readers with key takeaways. They should be commended for the time and effort that likely went into these high-level graphics. My comments below are minor.

In Table 2 (page 9) the column headers should be revised to better inform the reader of what is being presented. "% doctors" and "doctors as % of total" are utilizing different denominators; presenting them side-by-side may be confusing for readers – many of whom (reading this text) may not possess scientific backgrounds.

In Figure 2 (page 12) I would recommend changing the title to "Socioeconomic background of all X, stratified by year turned 18". I am not quite sure what "vs" means, and it was confusing to read at first pass.

Discussion
Overall, the discussion is fantastic. The findings are complex, and very nicely summarized. The paper could benefit from an expanded discussion of several items. My suggestions are suggestions only; I leave expansion of the discussion to the discretion of the editor and authors, as the section is already well-written.

a. Intersectionality – while the primary focus was SES, owing to powerful historical and contemporary forces like structural racism, race/ethnicity and SES are tightly linked. Race/ethnicity has been a primary focus of diversity efforts in the US (and abroad) for decades now. How do we reconcile SES diversity with racial/ethnic diversity among physicians? Discuss how race/ethnicity data was collected, stratified, and controlled for in this analysis. How does intersectionality in the UK differ from, for example, the US?
b. "Doctor households" – concerning the findings presented on page 13, lines 24-32. For decades it has been colloquial knowledge that many medical students have parents who are physicians. To my knowledge, this is the first time that this observation has been quantified and contrasted with (a) working class households, and (b) more privileged non-doctor backgrounds. Respondents from households with other professional occupations were still 8-fold less likely to be doctors. This observation merits discussion outside of "parental influence" alone.
c. Tools to address gaps – In the final paragraph (page 22), the authors may consider discussing tools available to medical school admissions departments in the UK to account for applicant SES in the admissions process. A nice analogy would be the use of the parental education and occupation (EO) indicator – a proxy available to US medical school admissions departments available through

the AMCAS (American Medical College Application Server) server, managed by the AAMC.

d. Finally, a high-level comparison of their observations with observations (to date) for the US medical education system

Minor (references)
Instead of citing the AAMC reports (references 15 and 16) the authors may consider citing our work examining the socioeconomic diversity of the matriculating medical student body where medical student SES was compared to population SES (Shahriar AA, Puram VV, Miller JM, et al. Socioeconomic Diversity of the Matriculating US Medical Student Body by Race, Ethnicity, and Sex, 2017 - 2019. *JAMA Netw Open*. 2022;5(3):e222621). The key finding, here, was the presence of stark socioeconomic diversity gaps across all racial-ethnic groups

---

**Reviewer**	**2**
**Name**	**Lambert, Paul**
**Affiliation**	**University of Stirling**
**Date**	**15-Apr-2025**
**COI**	**None**

Overall, a very strong analysis of secondary survey data is presented which adds valuable systematic evidence about social inequalities related to medical professions in the UK.

Good practice is shown in methodology, with transparent communication of processes, including commitment to documenting analysis code at Github. Missing data on social origins could present a risk to the evidence base, but careful steps are taken to confront this. Analytical results are presented in an engaging way, including some very well considered graphical summaries, and well-judged selective presentation of detailed results between main and appendix materials. The terms of analysis and context to the study are introduced well, and provide a compelling argument for research in the area. There are numerous useful connections with previous analyses of inequalities in access to professions in medicine.

There could be a case for minor adjustments to either the analysis or its framing in the following areas:

There's an argument for toning down the language of causality as used in the abstract. The analytical methods comprise Poisson models with various control variables. These provide complex descriptive evidence but are unlikely to provide convincing causal assessment, for instance given the plausibility of omitted variable bias and measurement error in controls. Measures of ability, for example, are candidates as sources of substantial omitted variable bias in the interpretation of socio-economic background coefficients.

Figures 5a and S2 seem to indicate very interesting patterns amongst control variables, and are based on quite a thoughtful arrangement of models as related to the DAG from Figure 1. Nevertheless, the relationship between country of birth and ethnicity in this system seems unconvincing sociologically – the two are more conventionally conceived as co-existing. As such the trend in ethnicity net of country of birth controls seems at risk of giving a misleading impression. For instance it could be that minority ethnicity has not increased in its impact on probability of being a doctor, just that it is less proxied by country of birth in younger cohorts.

Whilst interaction patterns amongst variables are due to be explored in later work, it does seem a limitation that they are not examined here in a little more depth (aside from via the stratification by year of birth). Two-way interactions between socio-economic background and gender, ethnicity or country of birth are all plausible, and brief assessment of them might be insightful to understanding inequalities.

In reflecting upon the contribution made, more connections with sociological studies of occupational inheritance might have some relevance. Reflection on efforts to study 'microclass' inequality, and to parameterise inheritance within social mobility models such as in the loglinear modelling tradition, could be insightful. Empirical comments on how occupational inheritance amongst doctors compares to other trades and professions, or indeed to other countries, would add value too – a few comparative statistics drawn from other studies might help the account here, but the rates amongst doctors in the UK are probably not particularly pronounced when compared to some other areas of work or other nations.

Lastly, the article concludes with thoughtful reflection on how much of a social problem the inequality that it identifies might be, and on what actions might follow. From a sociological framing, the BMA aspiration to representativeness as described seems rather naïve and might be assessed more critically. Philosophical reflections on whether 'perfect mobility' is desirable often conclude that it is more realistic to focus on interventions that respond to existing inequality patterns, rather than focussing on changing access probabilities. In the case of doctors in the UK, examples might involve seeking alternative mechanisms for doctor-patient interaction to confront any ill effects of mismatch in representation, or trying to curtail (rather than encourage) high pay and wealth growth amongst doctors.

VERSION 1 - AUTHOR RESPONSE

Reviewer: 1

Dr. Arman Shahriar, The University of Chicago

Comments to the Author:
Summary: In this observational study, the researchers assessed the effect of childhood socioeconomic status (SES) – as estimated by the occupation of the main household earner when the respondent was 14 years old – on ultimate occupation as a physician in the UK. The

methods are robust. The results are unique. The figures are excellent. The primary finding was a strong association between childhood SES and occupation as a physician. One important secondary finding was the magnitude of this observation among adolescents who hail from “doctor households” (11.5-13% probability; 15-fold more likely than those without physicians who are parents). The results are presented nicely in disaggregated fashion in Table 2 and summarized visually in Figure 2 – the key figure in this study. My comments are minor and stratified by section.

Methods

On page 5, line 49 the authors write:
“The exposures of interest were the occupation and socioeconomic status of the main earner of the household when respondent was aged 14”. As far as I can tell, socioeconomic status was entirely derived from occupation – which mapped to NS-SEC groups based on the Standard Occupational Classification system presented in Table 1. Thus, using the word “and” in the above sentence may be misleading. With that said, parental occupation has been validated as a proxy for SES in American literature (Grbic D et al Acad Med 2015; 90(7): 953-960).

We have edited the sentence to clarify that socio-economic status is directly derived from occupation.

Before: “The exposures of interest were the occupation and socioeconomic status of the main earner of the household when respondent was aged 14”

After: “The exposures of interest were the occupation and socioeconomic status **(derived from occupation)** of the main earner of the household when respondent was aged 14”

The missingness percentage (24%) is somewhat concerning, but most of us know from this sort of survey data, expected. Given the 24% missing data for a survey, I would recommend an assessment by a statistical reviewer (if other peer reviewers are not comfortable providing an assessment). They do discuss this in limitations (page 22)

No action required as 2nd reviewer brought in and their points addressed below

Results

The authors do a fantastic job of summarizing findings using appropriate visuals that can leave less scientific or even non-scientific readers with key takeaways. They should be commended for the time and effort that likely went into these high-level graphics. My comments below are minor.

In Table 2 (page 9) the column headers should be revised to better inform the reader of what is being presented. “% doctors” and “doctors as % of total” are utilizing different denominators; presenting them side-by-side may be confusing for readers – many of whom (reading this text) may not possess scientific backgrounds.

To improve clarity, we have renamed the columns

Before: % doctors

After: % doctors (as proportion of all current doctors)

Before: Doctors as % of total

After: % doctors (as proportion of respondents)

If editors can think of any other alternative wording that would be even clearer, that would be appreciated.

In Figure 2 (page 12) I would recommend changing the title to "Socioeconomic background of all X, stratified by year turned 18". I am not quite sure what "vs" means, and it was confusing to read at first pass.

We have made the suggested change to the titles within figure 2, to improve clarity.

Discussion

Overall, the discussion is fantastic. The findings are complex, and very nicely summarized. The paper could benefit from an expanded discussion of several items. My suggestions are suggestions only; I leave expansion of the discussion to the discretion of the editor and authors, as the section is already well-written.

a. Intersectionality – while the primary focus was SES, owing to powerful historical and contemporary forces like structural racism, race/ethnicity and SES are tightly linked. Race/ethnicity has been a primary focus of diversity efforts in the US (and abroad) for decades now. How do we reconcile SES diversity with racial/ethnic diversity among physicians? Discuss how race/ethnicity data was collected, stratified, and controlled for in this analysis. How does intersectionality in the UK differ from, for example, the US?

This is an important point from the reviewer that we appreciate.

We considered performing an intersectional analysis for this study, but decided to focus on socio-economic diversity as an individual characteristic, as it is relatively under-examined to-date, compared to others such as sex/gender and race/ethnicity. However, we acknowledge and understand that intersectional inequalities in entry to the medical profession are very important to examine in future, to understand how access to medicine varies across multiple characteristics such as sex/gender, race/ethnicity, and socio-economic background.

Therefore, to address this point, we have edited and expanded sentences emphasising the importance and need for future intersectional analysis as part of the limitations subsection of the discussion:

"We focused on socio-economic background as our main exposure due to current absence of comprehensive data collection, but acknowledge the importance of understanding how inequalities vary for different intersections of socio-economic background and other factors such as sex/gender and race/ethnicity, which should be expanded upon in future with further intersectional analysis. Furthermore, given the correlation between race/ethnicity and socio-economic status, resulting from complex histories of structural discrimination in many

countries across the world, it will be important to understand whether intersectional inequalities vary across countries, for example between the UK and USA."

Finally, to respond to the reviewer's question about how race/ethnicity data was collected, stratified, and controlled for in this analysis, we have expanded on these details in the methods section:

Concerning how data was collected:

"Age, sex, country of birth and ethnic group were self-reported from closed survey questions." (methods, Exposure and control variables subsection)

We also describe which categorisation of ethnic group we used later in the same paragraph:

"Response categories for the ethnic group question varied based on the respondent's nation of residence, and so the least detailed 9-category version common across UK nations was used". (methods, Exposure and control variables subsection)

We controlled for race/ethnicity in the analysis of socio-economic background, by including ethnic group as a variable within regression models. This is described in the first paragraph of the results subsection "Associations between working as a doctor and socio-economic background and occupation from multivariable models", as well as in relevant figure and table captions:

"Models controlled for year respondent turned 18 to control for trends in the relative shares of different occupations over time, as well as sex, country of birth, ethnic group and year of survey response."

b. "Doctor households" – concerning the findings presented on page 13, lines 24-32. For decades it has been colloquial knowledge that many medical students have parents who are physicians. To my knowledge, this is the first time that this observation has been quantified and contrasted with (a) working class households, and (b) more privileged non-doctor backgrounds. Respondents from households with other professional occupations were still 8-fold less likely to be doctors. This observation merits discussion outside of "parental influence" alone.

We agree this observation should be highlighted, and so we have added the following sentence to the end of the Interpretation and Strengths subsection of the Discussion:

"However, the extent to which doctor households remain an outlier among more privileged backgrounds is also noteworthy, with respondents from doctor households still 8-fold more likely to become doctors than those from non-doctor professional backgrounds (Table 3, model 3), and at least 3-fold more likely than any other detailed occupation group (Table 4, Figure 4)."

c. Tools to address gaps – In the final paragraph (page 22), the authors may consider discussing tools available to medical school admissions departments in the UK to account for applicant SES in the admissions process. A nice analogy would be the use of the parental education and occupation (EO) indicator – a proxy available to US medical school admissions departments available through the AMCAS (American Medical College Application Server) server, managed by the AAMC.

We thank reviewer for highlighting the EO indicator used in US medical school admissions. We have expanded the relevant sentence in the Limitations subsection of the Discussion to include this point:

Before:

"Finally, we note that the NS-SEC measure of occupational class is a single indicator of socio-economic status, and that collection of further indicators of socio-economic status during childhood, as outlined by Social Mobility Commission guidance, could provide a more detailed understanding of inequalities [49]."

After:

"Finally, we note that the NS-SEC measure of occupational class is a single indicator of socio-economic status, and that collection of further indicators of socio-economic status during childhood, as outlined by Social Mobility Commission guidance [49], or those used in other countries (e.g., the parental education and occupation indicator used by US medical schools [83]), could provide a more detailed understanding of inequalities among both medical students and current doctors."

[83] Grbic D, Jones DJ, Case ST. The Role of Socioeconomic Status in Medical School Admissions. Academic Medicine 2015;90:953–60. doi:10.1097/ACM.0000000000000653

d. Finally, a high-level comparison of their observations with observations (to date) for the US medical education system

To give as close a comparison as possible between UK and USA of the inequalities in socio-economic background among current doctors, we have added a sentence comparing proportions of current doctors from working class backgrounds to the "Interpretation & Strengths" subsection of the Discussion:

Before:

This inequality mirrors previous observations within medical school populations over the last 60 years in the UK [2,3,5,7] and internationally [10–17,19,20], but this analysis is the first to show the persistence and rigidity of these socio-economic inequalities among working doctors over a timespan of 50 years within a single, nationally representative sample

After:

This inequality mirrors previous observations within medical school populations over the last 60 years in the UK [2,3,5,7] and internationally [10–17,19,20]. **In the closest possible international like-for-like comparison, we found a slightly lower proportion of current doctors from working class backgrounds in the UK vs. USA – 14% vs. 20% (noting the US figure is based on a much smaller sample of 121 doctors whose socio-economic background was examined as part of a larger analysis of class pay differences in the 2010s) [57]. However, unlike other studies to-date**, this analysis is the first to show the persistence and rigidity of these socio-economic inequalities among working doctors over a timespan of 50 years within a single, nationally representative sample.

[57] Laurison D, Friedman S. The Class Ceiling in the United States: Class-Origin Pay Penalties in Higher Professional and Managerial Occupations. Social Forces 2024;103:22–44. doi:10.1093/SF/SOAE025

Minor (references)

Instead of citing the AAMC reports (references 15 and 16) the authors may consider citing our work examining the socioeconomic diversity of the matriculating medical student body where medical student SES was compared to population SES (Shahriar AA, Puram VV, Miller JM, et al. Socioeconomic Diversity of the Matriculating US Medical Student Body by Race, Ethnicity, and Sex, 2017 - 2019. JAMA Netw Open. 2022;5(3):e222621). The key finding, here, was the presence of stark socioeconomic diversity gaps across all racial-ethnic groups

We thank the reviewer for highlighting this work which makes useful comparison to population SES and stratification by racial-ethnic groups, and have added this work as an extra reference in the introduction.

Reviewer: 2

Dr. Paul Lambert, University of Stirling
Comments to the Author:

Overall, a very strong analysis of secondary survey data is presented which adds valuable systematic evidence about social inequalities related to medical professions in the UK.

Good practice is shown in methodology, with transparent communication of processes, including commitment to documenting analysis code at Github. Missing data on social origins could present a risk to the evidence base, but careful steps are taken to confront this. Analytical results are presented in an engaging way, including some very well considered graphical summaries, and well-judged selective presentation of detailed results between main and appendix materials. The terms of analysis and context to the study are introduced well, and provide a compelling argument for research in the area. There are numerous useful connections with previous analyses of inequalities in access to professions in medicine.

There could be a case for minor adjustments to either the analysis or its framing in the following areas:

There's an argument for toning down the language of causality as used in the abstract. The analytical methods comprise Poisson models with various control variables. These provide complex descriptive evidence but are unlikely to provide convincing causal assessment, for instance given the plausibility of omitted variable bias and measurement error in controls. Measures of ability, for example, are candidates as sources of substantial omitted variable bias in the interpretation of socio-economic background coefficients.

As suggested, we have changed causal language in the abstract, objectives subsection:

Before:

“To estimate the total causal effect of socio-economic background (derived from household main earner occupation when survey respondent was age 14) on likelihood of working as a doctor in adulthood in the UK, and estimate how effects varied over time for respondents who turned 18 years old in different decades.”

After:

“To estimate the **association between** socio-economic background (derived from household main earner occupation when survey respondent was age 14) on likelihood of working as a doctor in adulthood in the UK, and estimate how **associations** varied over time for respondents who turned 18 years old in different decades.”

Figures 5a and S2 seem to indicate very interesting patterns amongst control variables, and are based on quite a thoughtful arrangement of models as related to the DAG from Figure 1. Nevertheless, the relationship between country of birth and ethnicity in this system seems unconvincing sociologically – the two are more conventionally conceived as co-existing. As such the trend in ethnicity net of country of birth controls seems at risk of giving a misleading impression. For instance it could be that minority ethnicity has not increased in its impact on probability of being a doctor, just that it is less proxied by country of birth in younger cohorts.

We appreciate this point, and while we hypothesise that country of birth ‘crystallises’/is fixed at an earlier point in time than ethnic group (given that country of birth is objective and is set at birth, while ethnic group is a subjective self-identification that might change over time). However, we assume that for most people their ethnic group identity is set from early years, and the two variables remain highly correlated. So, we agree with the reviewer that controlling for country of birth in models estimating associations with ethnic group may be misleading. Therefore, we have removed country of birth as a control variable in regression models estimating association with ethnic group (Figure 5(a), Figure S1, Figure S2).

We have revised the description of the time-series analysis results in the “Models stratified by year turned 18” subsection of the Results accordingly:

Before:

The higher likelihood of respondents born outside of the UK to be doctors in comparison to those born in the UK fell twice, once in the 1960s, and again between 1985 and 2000. From 2000 onwards, respondents born outside of the UK remained 1.5 to 2-fold more likely to be doctors. Conversely, respondents from racially minoritised ethnic groups (Asian/Asian British, Black/Black British, Mixed/Multiple ethnic groups, and Any other ethnic groups) became increasingly more likely than White ethnic group respondents to be doctors among those reaching adulthood between 1980 and 2000 (models controlling for country of birth). From 2000 onwards, respondents from racially minoritised ethnic groups remained 3 to 4-fold more likely to be doctors. Results for disaggregated country of birth and ethnic group variables are given in Figure S2.

After:

The higher likelihood of respondents born outside of the UK to be doctors in comparison to those born in the UK fell twice, once in the 1960s, and again between 1985 and 2000. From 2000 onwards, respondents born outside of the UK remained 1.5 to 2-fold more likely to be doctors.

Similarly, the higher likelihood of respondents from racially minoritised ethnic groups (Asian/Asian British, Black/Black British, Mixed/Multiple ethnic groups, and Any other ethnic groups) to be doctors in comparison to White ethnic group respondents showed an initial fall in the 1960s, then remained among 3 to 4-fold more likely from 1970 onwards (in models not controlling for country of birth). Results for disaggregated country of birth and ethnic group variables are given in Figure S2.

Whilst interaction patterns amongst variables are due to be explored in later work, it does seem a limitation that they are not examined here in a little more depth (aside from via the stratification by year of birth). Two-way interactions between socio-economic background and gender, ethnicity or country of birth are all plausible, and brief assessment of them might be insightful to understanding inequalities.

We agree that further assessment of interactions between multiple socio-demographic variables would be insightful, and we contemplated performing an intersectional analysis to examine this. However, we decided to focus on socio-economic diversity as an individual characteristic, as it is relatively under-examined to-date, compared to others such as sex/gender and race/ethnicity, and wanted this to be the key focus of this study.

Following a similar point made by Reviewer 1, we have expanded discussion of this point in the Limitations subsection of the Discussion – please see details above in our response to Reviewer 1 Discussion comment (a). We plan to follow this work with a further study which focuses solely on looking at these interactions and intersectional inequalities, in a way that we think wouldn't be possible to do fully in this manuscript given space constraints.

In reflecting upon the contribution made, more connections with sociological studies of occupational inheritance might have some relevance. Reflection on efforts to study 'microclass' inequality, and to parameterise inheritance within social mobility models such as in the loglinear modelling tradition, could be insightful. Empirical comments on how occupational inheritance amongst doctors compares to other trades and professions, or indeed to other countries, would add value too – a few comparative statistics drawn from other studies might help the account here, but the rates amongst doctors in the UK are probably not particularly pronounced when compared to some other areas of work or other nations.

We thank the reviewer for this comment, as it is important to expand and give context of how microclass reproduction within medicine compares to other occupations. To do this, we've made reference to studies looking at microclass reproduction in the UK, where doctors had the highest intergenerational reproduction among 'elite' occupations, and a study of social mobility in other high income nations, where doctors also had reproduction coefficients among the highest for higher income occupations.

The following sentence has been added at the end of the 'Strengths & Interpretation' subsection of the discussion:

"In the wider context of occupational reproduction across generations, doctors have been found to have the highest level of so-called 'microclass reproduction' in analysis of workers in 2010s UK [34]. Offspring of doctors were much more likely to work in the same occupation as their parents/carers than other high income professions such as law, architecture or finance.

Similarly, social mobility analysis of data from 1962-2002 in Germany, Japan, Sweden and USA, found doctors had the highest microclass reproduction after airline pilots, ship officers, and clergy [60].”

References:

[34] Friedman S, Laurison D. The Class Ceiling: Why it Pays to be Privileged. Policy Press 2019 (Page 35)

[60] Jonsson JO, Grusky DB, Di Carlo M, et al. Microclass Mobility: Social Reproduction in Four Countries. American Journal of Sociology 2009;114:977–1036. doi:10.1086/596566

Lastly, the article concludes with thoughtful reflection on how much of a social problem the inequality that it identifies might be, and on what actions might follow. From a sociological framing, the BMA aspiration to representativeness as described seems rather naïve and might be assessed more critically. Philosophical reflections on whether ‘perfect mobility’ is desirable often conclude that it is more realistic to focus on interventions that respond to existing inequality patterns, rather than focussing on changing access probabilities. In the case of doctors in the UK, examples might involve seeking alternative mechanisms for doctor-patient interaction to confront any ill effects of mismatch in representation, or trying to curtail (rather than encourage) high pay and wealth growth amongst doctors.

We agree with reviewer that focusing solely on inequalities in entry to medicine is likely unrealistic and may be the wrong focus given that there is currently a lack of evidence as to whether discordance, in terms of socio-economic background, between patient and doctor is associated with patient experience. However, this is due to absence of studies with this focus, rather than no evidence of association. Therefore, to further emphasise the need to understand the role of mismatches in representation in doctor-patient interactions and patient experiences, we have added the following sentence at the end of the “Implications for clinicians and policymakers” subsection of the Discussion:

“However, given the apparent stubbornness of inequalities in access to medicine, it may be more useful to shift focus to understanding and addressing any repercussions for patients of socio-economic inequality among doctors. This may include studying whether socio-economic concordance in patient-doctor dyads affects patient-rated experiences, and/or developing ‘cultural competence’ training of practising doctors to share learnings from previous studies detailing the needs of lower socio-economic status patients from patient-doctor interactions [64,65].”

Other changes

1. We have updated values for adjusted mean predicted probabilities of being a doctor (presented in Table 3, Table 4, Table S1) after realising the method previously used was not best practice while the article was under review. Consequently, we used a different software package, which is detailed in methods.

Predicted probabilities have been revised wherever they are quoted within the main text prose, and in tables - main text Table 3 & Table 4, supplementary information Table S1. As the ordering

of rows in Table 4 and Table S1 is based on the mean predicted probability values, some rows have changed order slightly due to small changes in probabilities. However, the trends and interpretation remain the same.

Changes in the methods highlighted below:

Before: “Mean predicted probabilities of being a doctor were also generated from multivariable regression models by calculating the mean of individual predicted probabilities of all respondents in the given category of interest and the mean of the 95% confidence intervals.”

After: “Adjusted mean predicted probabilities of being a doctor were generated from regression model fits following an ‘average marginal effects’ or ‘marginal standardisation’ approach [54], using the “marginaleffects” R package [55]. In this approach, individual level predicted probabilities were generated after assigning all individuals to a single category of the exposure variable, while keeping all other model variables at their observed values. The mean of all predicted probabilities was then calculated, while accounting for inverse probability weights. This process was repeated while varying the exposure variable value, thereby generating a mean predicted probability for all exposure categories. Again, 95% confidence intervals were calculated using the “HC3” estimator.”

2. We have also added a reference to the introduction and the limitations subsection of the discussion to a recent study that came out while the article was under review, about recent socio-economic diversity among medical students in the UK.

3. We have added a ‘software’ subsection to the methods, to detail which software and packages were used in the analysis.

VERSION 2 - REVIEW

**Reviewer**	**1**
**Name**	**Shahriar , Arman**
**Affiliation**	**The University of Chicago, Internal Medicine**
**Date**	**21-May-2025**
**COI**	

Thank you for making thoughtful revisions to reflect the majority of the points I had raised. Congratulations on your excellent work.
